# Formation of compounds with diverse polyelectrolyte morphologies and nonlinear ion conductance in a two-dimensional nanofluidic channel†

**DOI:** 10.1039/d4sc01071j

**Published:** 2024-05-03

**Authors:** Xiaoying Liang, Yanan Zhou, Weiduo Zhu, Wen Wu Xu, Joseph S. Francisco, Xiao Cheng Zeng, Wenhui Zhao

**Affiliations:** a Department of Physics, Ningbo University Ningbo Zhejiang 315211 China zhaowenhui@nbu.edu.cn; b School of Material Science and Chemical Engineering, Institute of Mass Spectrometry, Ningbo University Ningbo 315211 China; c Department of Physics, Hefei University of Technology Hefei Anhui 230009 China; d Department of Chemistry, University of Pennsylvania Philadelphia Pennsylvania 19104 USA frjoseph@sas.upenn.edu; e Department of Materials Science & Engineering, City University of Hong Kong Kowloon 999077 Hong Kong China xzeng26@cityu.edu.hk

## Abstract

Aqueous electrolytes subjected to angstrom-scale confinement have recently attracted increasing interest because of their distinctive structural and transport properties, as well as their promising applicability in bioinspired nanofluidic iontronics and ion batteries. Here, we performed microsecond-scale molecular dynamics simulations, which provided evidence of nonlinear ionic conductance under an external lateral electric field due to the self-assembly of cations and anions with diverse polyelectrolyte morphologies (*e.g.*, extremely large ion clusters) in aqueous solutions within angstrom-scale slits. Specifically, we found that the cations and anions of Li_2_SO_4_ and CaSO_4_ formed chain-like polyelectrolyte structures, whereas those of Na_2_SO_4_ and MgSO_4_ predominantly formed a monolayer of hydrated salt. Additionally, the cations and anions of K_2_SO_4_ assembled into a hexagonal anhydrous ionic crystal. These ion-dependent diverse polyelectrolyte morphologies stemmed from the enhanced Coulomb interactions, weakened hydration and steric constraints within the angstrom-scale slits. More importantly, once the monolayer hydrated salt or ionic crystal structure was formed, the field-induced ion current exhibited an intriguing gating effect at a low field strength. This abnormal ion transport was attributed to the concerted movement of cations and anions within the solid polyelectrolytes, leading to the suppression of ion currents. When the electric field exceeded a critical strength, however, the ion current surged rapidly due to the dissolution of many cations and anions within a few nanoseconds in the aqueous solution.

## Abstract 2

Our molecular dynamics simulations revealed diverse polyelectrolyte morphologies in angstrom-scale slits, resulting in intriguing gating effects and nonlinear ionic conductance.

## Graphical abstract

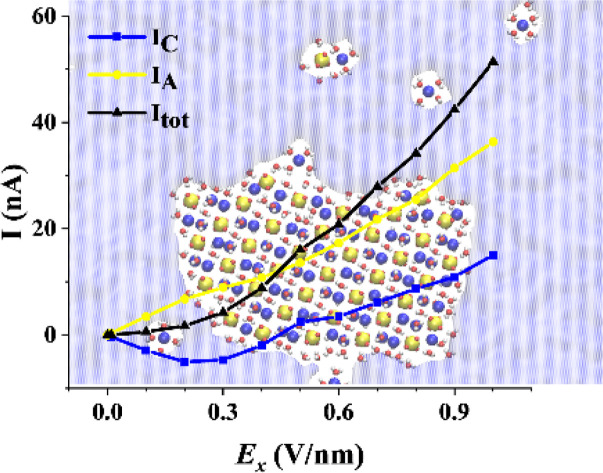

## Introduction

In recent years, there has been increasing interest in aqueous solutions subjected to nanoscale confinement due to their unique structural and dynamical properties, as well as their potential applicability in fields such as bioinspired nanofluidic iontronics, ion batteries and desalination.^1–6^ Recent studies have demonstrated that when this confinement reaches a critical length of approximately 1 nm, traditional bulk-fluid hydrodynamics breaks down.^7^ According to Lydéric Bocquet and coworkers,^8^ below 10 nm—the realm of single-digit nanopores—thermal fluctuations and electrostatic correlations become increasingly significant, posing challenges to continuum and mean-field theories. In confinements of just a few nanometers, fluid structuring effects and correlations play an overwhelmingly important role. For instance, water molecules can adopt polygonal prism configurations in quasi-one-dimensional (Q1D) nanotubes, even forming a single-file chain within a (6, 6) carbon nanotube with a diameter of 0.81 nm.^9–11^ Furthermore, in quasi-two-dimensional (Q2D) nanoslits, water molecules form layered structures.^12–14^ These structured water configurations lead to anomalous transport phenomena in the framework of nanoscale confinement.^15–18^ Therefore, comprehending the structural features of aqueous solutions subjected to nano- and angstrom-scale confinement is of fundamental importance for increasing our understanding of these nanoscale systems and their practical applications.

Compared with nanoconfined water, aqueous electrolytes subjected to nano-/angstrom-scale confinement exhibit more unusual and intriguing behaviours.^19,20^ These new structural and transport properties stem from the intricate interplay of various interactions, including long-range electrostatic forces, van der Waals interactions, ion hydration and/or dehydration, hydrogen bonding, steric effects, *etc.*^21–23^ Nanoconfinement enhances the Coulomb interactions between ions compared to those in the bulk fluid,^24,25^ as the dielectric constant of confined aqueous solutions is considerably decreased relative to that of nonconfined solutions.^26,27^ As a result, novel phenomena can emerge, such as ionic Coulomb blockade,^22,28^ loss of electroneutrality,^29^ ion-exchange phase transitions,^30^ and exceptionally high ion diffusivity.^5^

Very recently, based on nanosecond-scale molecular dynamics (MD) simulations, Bocquet and coworkers made the first prediction of highly nonlinear phenomena during ion transport through angstrom-scale slits.^31^ These unique properties of nanostructured aqueous solutions resulted from the formation of tightly bound Bjerrum ion pairs within a range of 15 ns. Furthermore, their theoretical study revealed that ion pairs can reorganize into Bjerrum polyelectrolyte-like aggregates under an external electric field, leading to hysteresis in field-induced ion conduction similar to the memristor effect. Later, these theoretical predictions were experimentally confirmed. Specifically, these researchers successfully realized 2D nanofluidic ionic memristors with an exceptionally extended memory timescale spanning from seconds to hours.^32^ This groundbreaking work represents a major advance in nanofluidic iontronics and the emerging field of neuromorphic ionic computing.^33–36^

In our recent work, we comprehensively investigated the phase behaviour of aqueous electrolyte solutions in 2D angstrom-scale slits^37–39^ using microsecond-scale MD and Born–Oppenheimer molecular dynamics (BOMD) simulations. We found that even in the absence of an electric field, spontaneous ion aggregation into polyelectrolyte-like clusters occurred within several hundred nanoseconds or even a few microseconds. In the case of alkali metal chloride aqueous solutions, we showed that Na^+^ and Cl^−^ ions self-assembled into a monolayer anhydrate ionic crystal with a square-unit pattern. In contrast, Li^+^ and Cl^−^ ions formed disordered hexagonal ring and/or chain patterns featuring Bjerrum ion pairs.^37^ Furthermore, alkaline-earth metal chlorides, including MgCl_2_, CaCl_2_, BaCl_2_ and SrCl_2_, tended to aggregate into monolayer hexagonal honeycomb hydrated salts.^39^

Despite significant research efforts, the structural and transport properties of aqueous electrolyte solutions in angstrom-scale slits remain incompletely understood due to the diverse types of ions formed with different elements, valence states and sizes.^23,25,34^ In this study, we systematically investigated the impact of ion valence states and sizes on the self-assembly of sulfate electrolyte aqueous solutions within angstrom-scale slits using microsecond-scale MD simulations. Additionally, we examined the effects of structure on ion transport under the influence of an external electric field. We considered several prototypical cations commonly found in seawater and biological fluids, namely, the monovalent cations Li^+^, Na^+^ and K^+^ and the divalent cations Mg^2+^ and Ca^2+^. Our focus on the sulfate ion (SO_4_^2−^) was because it is a prototype divalent anion, and it possesses unique properties compared to monovalent anions like chloride ions (Cl^−^ ions). Additionally, the high solubility of sulfate salts, such as sodium sulfate and magnesium sulfate, in water reinforced our selection of the sulfate electrolyte aqueous solutions for this investigation. Depending on the valence states and sizes of the cations, we observed spontaneous self-assembly of cations and sulfate anions (SO_4_^2−^) into four distinct monolayer polyelectrolyte structures, each featuring a unique structural pattern. These patterns included disordered chains (Li_2_SO_4_ and CaSO_4_), two types of monolayer hydrated salts (rhomboidal 4Na·2SO_4_·6H_2_O and square MgSO_4_·6H_2_O), and a hexagonal honeycomb K_2_SO_4_ anhydrate ionic crystal. Notably, the disordered chains of Li_2_SO_4_ and CaSO_4_ consisted of hydrated ions, in contrast to the disordered chains of LiCl, which were composed of Bjerrum ion pairs.^37^ Importantly, we highlighted the presence of a nonlinear effect during the ion transport of the five aqueous electrolyte solutions through angstrom-scale slits when subjected to an external electric field. Interestingly, for the monolayer hydrated salt and ionic crystal structures, we observed a gating effect on the ion current at a low electric field, as the polyelectrolytes facilitated the coordinated movement of cations and anions, resulting in suppression of the currents. When the electric field exceeded a specific threshold, the ion current increased more rapidly due to the greater number of ions dissolving into the aqueous solution within the nanosecond timescale.

## Results and discussion

### Self-assembly of monolayer polyelectrolyte-like ion clusters of alkali sulfates

**Fig. 1:**
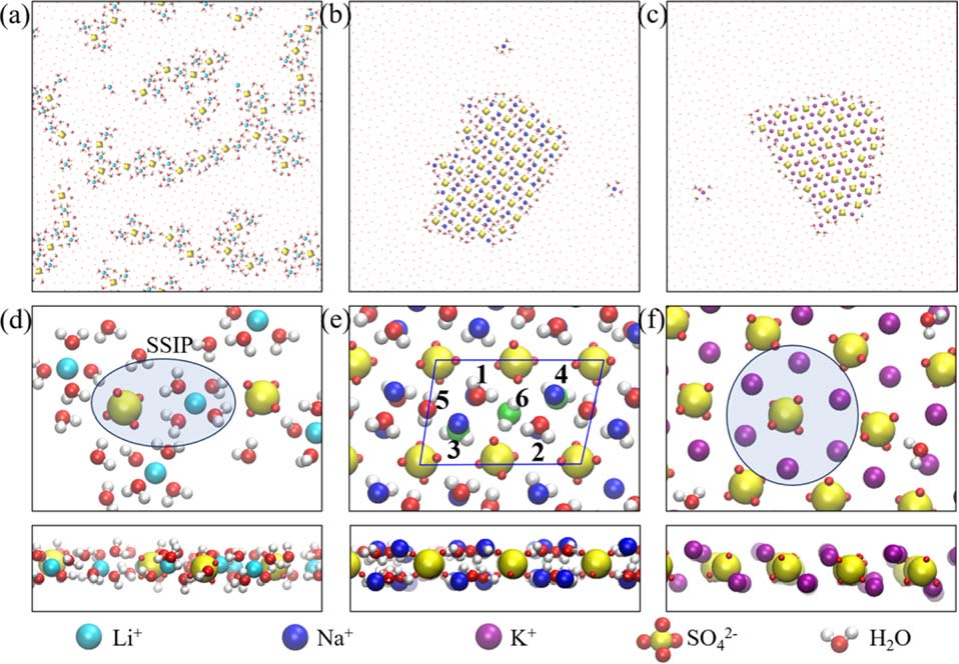
Monolayer polyelectrolytes of alkali sulfates in a nanoslit with width *D* = 0.8 nm. Top views of snapshots of (a) Li_2_SO_4_ polyelectrolyte-like chains, (b) a 4Na·2SO_4_·6H_2_O hydrated salt, and (c) a K_2_SO_4_ ionic crystal. (d–f) The corresponding magnified top and side views of a portion of the snapshots (shown in (a)–(c)) highlighting hydration or dehydration. The section highlighted in Fig. 1d indicates a solvent-separated ion pair (SSIP), while the highlighted portion in Fig. 1f indicates full dehydration. The blue rhombus in Fig. 1e indicates the rhomboidal unit cell with four Na^+^ cations, two SO_4_^2−^ anions and six water molecules. The digits 1–6 indicate the six water molecules included in the unit cell, and the green spheres represent the oxygen atoms of the water molecules adjacent to the bottom wall.

We first carried out microsecond-scale MD simulations of alkali sulfate (Li_2_SO_4_, Na_2_SO_4_ or K_2_SO_4_) aqueous electrolyte solutions confined between two smooth hydrophobic plates with a width of 0.8 nm. The system consisted of 1222 water molecules, 40 anions (SO_4_^2−^), and 80 monovalent cations or 40 divalent cations (see Methods for details). The dynamic trajectories showed that during the initial few nanoseconds of the three independent simulations, notably disordered polyelectrolyte-like ion clusters formed (ESI Movies S1–S3†). Next, after 4–7 μs of equilibration, distinct monolayer ion aggregates formed spontaneously. Note that here the self-assembly of the polyelectrolytes occurred even in the absence of an external electric field. In a previous study, however, the formation of the “Bjerrum polyelectrolytes” required an external electric field.^31^ The first type of aggregate consisted of polyelectrolyte-like chains spontaneously formed in an aqueous Li_2_SO_4_ solution (Fig. 1a). Note that in contrast to the Bjerrum polyelectrolyte-like chains in LiCl, which were composed of contact ion pairs (CIPs),^37^ the Li_2_SO_4_ polyelectrolyte-like chains consisted of solvent-separated ion pairs (SSIPs), as illustrated in Fig. 1d.

The second type of polyelectrolyte, namely, a monolayer hydrated salt with a distinctive rhomboidal pattern, denoted 4Na·2SO_4_·6H_2_O, formed as a result of the spontaneous aggregation of Na^+^ and SO_4_^2−^ ions (see Fig. 1b and e). Notably, this pattern differs from the monolayer hexagonal honeycomb hydrated salts formed by alkaline-earth chlorides.^39^ As depicted in Fig. 1e, this monolayer rhomboidal hydrated salt contained four water-Na^+^ pairs at the positions denoted 1, 2, 3 and 4 in Fig. 1e, that is, the four water-Na^+^ pairs were in registry, whereas the other two water molecules were not associated with Na^+^ ions and were situated at positions 5 and 6 in Fig. 1e. Note that positions 1, 2 and 5 are adjacent to one wall, whereas 3, 4, and 6 are adjacent to another (opposite) wall. The oxygen atoms of the water molecules at the latter three positions are highlighted as green spheres in Fig. 1e. Hence, the unit cell of the 4Na·2SO_4_·6H_2_O hydrated salt consisted of 4 Na^+^ ions and 2 SO_4_^2−^ ions and 6 water molecules, unlike the Na_2_SO_4_·3H_2_O hydrated salt. The newly discovered unit cell configuration of 4Na·2SO_4_·6H_2_O results in charge neutrality by accommodating mixed monovalent cations and divalent anions.

The third type of polyelectrolyte formed as a result of the spontaneous aggregation of K^+^ and SO_4_^2−^ ions into a monolayer ionic crystal, which included only a few water molecules that served as interstitial defects (see Fig. 1c). In contrast to the square pattern of the monolayer NaCl ionic crystal,^37^ the monolayer K_2_SO_4_ ionic crystal exhibited a hexagonal honeycomb pattern. Notably, the SO_4_^2−^ ion was slightly off-centre within the hexagon formed by six K^+^ ions (Fig. 1f). This off-centre positioning resulted from the asymmetrical Coulomb interaction between K^+^ ions and SO_4_^2−^ ions and the tetrahedral structure of SO_4_^2−^ ions in contrast to the spherical structure of Cl^−^ ion.

To gain insight into the distinctive structural characteristics of the three types of monolayer polyelectrolyte-like aggregates, we calculated the lateral ion-water (O atom) and ion–ion radial distribution functions (RDFs) in *xy* plane (ESI Fig. S1 and S2†). For the Li_2_SO_4_ polyelectrolyte, the sharp first peak followed by much broader and lower peaks in the lateral RDFs indicated disordered behaviour (Fig. S1a and S2a†). The prominent first peak of the Li-OW RDF, located at ∼0.26 nm, indicated the formation of a hydration shell around each Li^+^ ion. Likewise, the first peak at ∼0.47 nm of the Li–S RDF indicated the presence of hydrated Li^+^ ions near the SO_4_^2−^ ions. The coordination number (CN) of water molecules surrounding the Li^+^ ion was found to be 4 (Fig. S1d†), similar to the structure observed in the bulk. However, as illustrated in Fig. 1d, the hydration shells within the angstrom-scale slit exhibited a flattened configuration, resulting in reduced ion hydration interactions.

The 4Na·2SO_4_·6H_2_O and K_2_SO_4_ polyelectrolytes displayed a high degree of structural order, and their RDFs were characterized by well-defined density maxima and minima (Fig. S1b, c and S2b, c†). However, the two ordered polyelectrolytes still exhibited notable differences. The 4Na·2SO_4_·6H_2_O polyelectrolyte contained water molecules, whereas the K_2_SO_4_ polyelectrolyte formed a water-free ionic crystal. As displayed in Fig. S1b (ESI),† the pronounced first peak of the Na-OW RDF was located at approximately 0 nm, corresponding to an in-registry arrangement in which Na^+^ ions were associated with water molecules, as shown by the water molecules at positions 1, 2, 3 and 4 in Fig. 1e. This arrangement was further demonstrated by the second peak of the Na-OW RDF and the first peak of the Na–Na RDF at ∼0.23 nm. Here, the second peak of the Na-OW RDF indicated the presence of two water molecules, one associated with a Na^+^ ion (position 3 in Fig. 1e) and one not associated with a Na^+^ ion (position 6 in Fig. 1e). Similarly, the third peak of the Na-OW RDF at ∼0.35 nm corresponded to another water molecule, at position 5 in Fig. 1e, that was not associated with Na^+^ ions. Consequently, the coordination number (CN) of water molecules surrounding a Na^+^ ion was approximately 3 (Fig. S1e†), even though the unit cell of the 4Na·2SO_4_·6H_2_O monolayer polyelectrolyte contained 6 water molecules. This unit-cell configuration reflected the partial dehydration of ions and the subsequent reconfiguration of their hydration shells, which were notably compressed. For the K_2_SO_4_ ionic crystal, there was a small peak following the prominent first peak of the K–S RDF, consistent with the fact that the SO_4_^2−^ ion was slightly off-centre within the hexagonal arrangement of six K^+^ ions (Fig. 1f).

### Nucleation mechanism of monolayer polyelectrolytes of alkali sulfates

**Fig. 2:**
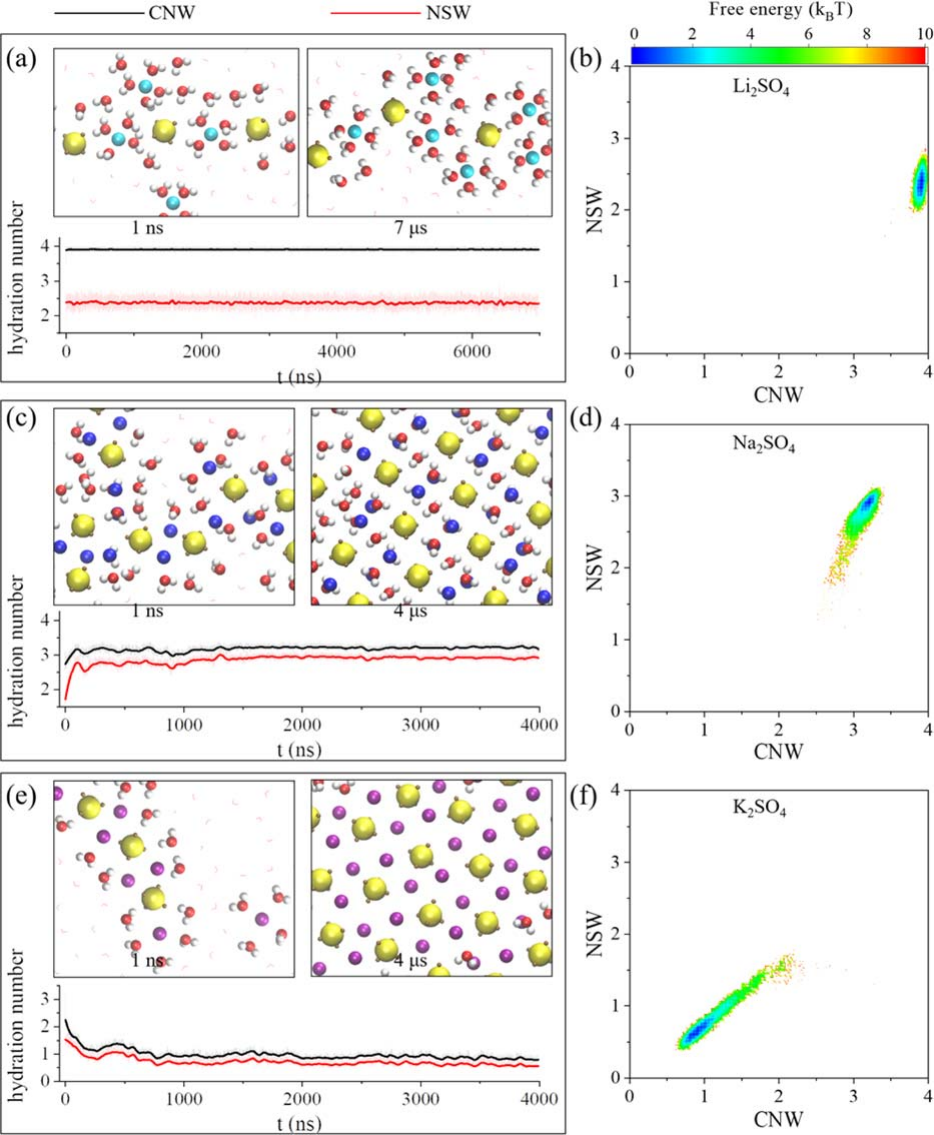
Nucleation mechanism of monolayer polyelectrolytes of alkali sulfates. The time-dependent average coordination number of water molecules (CNW) per cation and the number of shared water molecules (NSW) between cations and anions, computed based on representative snapshots during the 4–7 μs simulations of (a) Li_2_SO_4_, (c) Na_2_SO_4_ and (e) K_2_SO_4_. (b, d and f) The corresponding 2D conformational free energy landscapes.

To characterize the fundamental processes underlying the nucleation and growth of specific monolayer polyelectrolytes within angstrom-scale slits, we recorded the temporal evolution of the CN of water molecules in the solvation shells surrounding ions (Fig. 2). Moreover, the representative snapshots of polyelectrolyte domains extracted from various stages of the MD simulations were also displayed. These analyses can provide valuable insights into the dynamic behavior and structural organization of the polyelectrolyte assemblies during nucleation and growth processes.

Specifically, we analysed the time-dependent average coordination number of water molecules per cation (CNW) and the time-dependent average number of shared water molecules between cations and anions (NSW) based on the first peaks in the lateral RDFs. Here, the CNW defined as the average number of water molecules in the first hydration shell of the cation. The shared water molecule referred to a water molecule existing in the first hydration shells of both the cation and anion, and the NSW represented the average number of shared water molecules per cation. Importantly, the count included shared water molecules in the first hydration shells of different anions.

As shown in Fig. 2a, the average hydration number of Li^+^ ions remained ∼4 throughout the 7 μs simulation. Although this hydration number was nearly the same as that in the bulk solution (Fig. S3a†), the solvation shell of the Li^+^ ion exhibited a flattened configuration due to confinement, resulting in reduced ion hydration interactions. Moreover, the shared water number was ∼2.5, which was markedly smaller than the CNW (*i.e.*, 4). This finding suggested that the extremely large clusters corresponded to chain-like polyelectrolytes rather than a sheet-like structure. Furthermore, the statistics related to the nucleation events and growth of the polyelectrolytes through the 2D conformational free-energy landscapes, as determined according to −*k*_B_*T* ln *P*(CNW, NSW), are shown in Fig. 2b. Here, *k*_B_ represents Boltzmann's constant, and *P*(CNW,NSW) is the 2D probability density of CNW and NSW. The unimodal behaviour in the free-energy landscape indicated that the chain-like polyelectrolytes of Li_2_SO_4_ were the most stable. This outcome was attributed to the reduced ion hydration interaction and enhanced Coulomb interaction within the angstrom-scale slit.

As shown in Fig. 2c, during the initial few nanoseconds of the simulation, a disordered polyelectrolyte of Na^+^ and SO_4_^2−^ ions formed as a result of the enhanced Coulomb interactions within the angstrom-scale slit. Note that the ions within the polyelectrolyte were only partially hydrated by water molecules. Consequently, both the CNW and NSW values were much smaller than those observed for the Li_2_SO_4_ system, where the SSIPs exhibited a fully hydrated structure with a flattened solvation shell. This concurrent decrease in both the CNW and the NSW was a direct consequence of the formation of a 4Na·2SO_4_·6H_2_O monolayer hydrated salt. These findings were consistent with the characteristics discussed earlier, wherein each Na^+^ ion was hydrated by three water molecules that were also coordinated to SO_4_^2−^ ions.

The 2D conformational free-energy landscape further illustrated the stability of 4Na·2SO_4_·6H_2_O monolayer hydrated salt, coinciding with an increase in both the CNW and the NSW (Fig. 2d). Conversely, the lower values of the CNW and NSW for K_2_SO_4_ indicated that the first several hundred nanoseconds of the K_2_SO_4_ simulation involved dehydration processes (Fig. 2e). Eventually, the CNW and NSW values approached small constants (<1), indicating the formation of anhydrate polyelectrolytes of K_2_SO_4_ (Fig. 2f).

Comparative analysis with data of bulk electrolyte solutions (Fig. S3†) over 20 ns simulations revealed consistent hydration dynamics. The hydration numbers for Li^+^, Na^+^, and K^+^ ions were approximately 4, 5.4, and 6.3, respectively, suggesting no dehydration occurred. Moreover, the presence of structured solvation environments, indicated by NSW values ranging from 0.8 to 1.8, confirmed the formation of solvent-separated ion pairs (SSIPs) in bulk electrolyte solutions. The results further demonstrated that the formation of the monolayer polyelectrolytes was induced by the nanoconfinement.

The conformational free-energy landscapes provided additional insight into the hydration levels of the ions. The Li^+^ ions were fully hydrated, as evidenced by the hydration number CNW of ∼4 (Fig. 2b). In contrast, the Na^+^ ions experienced partial dehydration (Fig. 2d), and the K^+^ ions were fully dehydrated (Fig. 2f). These results followed the pattern of K^+^ > Na^+^ > Li^+^, opposite to the order of hydration energy: K^+^ < Na^+^ < Li^+^. Indeed, the hydration energy depended on the size of ions with same valence state; smaller ions (Li^+^) had higher hydration energy, whereas larger ions (K^+^) had lower hydration energy. The ion hydration interaction was diminished within the angstrom-scale slit due to the effects of dehydration and rearrangement of water molecules around the ions. Hence, the diverse assembly behaviours of Li_2_SO_4_, Na_2_SO_4_, and K_2_SO_4_ in the nanoslit with 0.8 nm width manifested their distinct hydration characteristics under subnanoscale confinement.

Additionally, we carried out MD simulations to examine effect of nanoslit width on the assembly behaviour. As shown in Fig. S4,† for nanoslit with a 0.75 nm width, the Li_2_SO_4_ and K_2_SO_4_ also adopted chain-like polyelectrolyte and anhydrate ionic crystal structure, respectively, as in nanoslit with 0.8 nm width. However, the in-registry hydrated salt of Na_2_SO_4_ was not observed. In other words, the stronger confinement induced the out-of-registry Na_2_SO_4_ hydrated salt (Fig. S4b and e†). In nanoslit with 0.85 nm width, the Li_2_SO_4_ also exhibited polyelectrolyte chains with hydrated ions (Fig. S5a and d†). However, the Na_2_SO_4_ hydrated salt in this case incorporated more water molecules (Fig. S5b and e†). More interestingly, the cations and anions of K_2_SO_4_ assembled spontaneously into a monolayer hydrated salt with a rhomboidal pattern for 4K·2SO_4_·6H_2_O (Fig. S5c and f†), identical to the case of Na_2_SO_4_ in a nanoslit with 0.8 nm width. These results further demonstrated that the formation of various polyelectrolytes is a manifestation of the delicate interplay among enhanced Coulomb interactions, weakened hydration, and strong steric constraint within angstrom-scale slits.

Specifically, nanoconfinement rendered the effect of Coulomb interactions stronger and diminished hydration, thereby promoting ion aggregation in aqueous solutions. Furthermore, hydration and steric constraints, influenced by ion size, dictated the type of self-assembly. For instance, smaller Li^+^ ions formed fully hydrated polyelectrolyte-like chains, while mid-sized Na^+^ ions underwent partial hydration. Nanoconfinement induced the rearrangement of water molecules, resulting in in-registry hydrated salt formation of Na_2_SO_4_ in nanoslit with 0.8 nm width, and out-of-registry Na_2_SO_4_ hydrated salt in nanoslit with 0.75 nm width. Conversely, larger K^+^ ions were fully dehydrated in nanoslit with 0.8 nm or 0.75 nm width but partially hydrated in nanoslit with 0.85 nm width.

Furthermore, to gain insight into the concentration dependence of the self-assembling behavior, we extended our simulation to encompass much more dilute solutions, with only 10 anions and 20 monovalent cations. Interestingly, even in these highly diluted scenarios, we observed consistent self-assembling behaviors (Fig. S6†). This observation underscores the generality of the self-assembly phenomena across a wide range of electrolyte concentrations.

The self-assembly of electrolytes in 2D nanofluid channels revealed substantial ionic correlation that was often overlooked with the conventional Nernst–Einstein approach. To accurately analyze the transport properties of such systems, we employed the theory of correlated systems.^40–46^ Using the position correlation function (PCF), we determined the symmetric mass-transport matrix *L* and the correlated transference number *t* (see Methods for details). Our findings, illustrated in Fig. S7† and Table 1, showed predominantly positive *L*_CA_ values across all five systems. This result suggested a positive correlation between cations and anions, indicating that they tended to move in similar directions on average. Notably, higher positive values for Na_2_SO_4_, K_2_SO_4_, and MgSO_4_ suggest stronger correlations, consistent with the formation of hydrated salt or ionic crystal structures. Additionally, the near-zero ionic conductivity *κ* further demonstrated that few free ions and charged small clusters were dissolved in the solution of the hydrated salt or ionic crystal system.

**Table 1:** The values of all the entries of the 
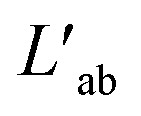
 matrix, the ionic conductivity *κ*′ and transference number (*t*) for Li_2_SO_4_, Na_2_SO_4_, K_2_SO_4_, MgSO_4_, and CaSO_4_ systems. C and A represented the cation and anion, respectively

	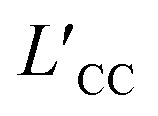	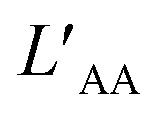	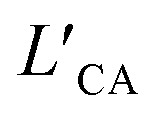	*κ*′	*t*_C_	*t*_A_
Li_2_SO_4_	5.99	6.13	5.32	1.48	0.453	0.547
Na_2_SO_4_	171	173	172	0	—	—
K_2_SO_4_	157	157	157	0	—	—
MgSO_4_	53.7	53.7	53.7	0	—	—
CaSO_4_	4.81	4.84	4.32	1.01	0.485	0.515

Furthermore, based on the obtained dynamic trajectories, coupled with the findings by Robin *et al.*,^31^ we found that the aggregates remained stable over much longer time scales (on the order of microseconds) than typical molecular time scales. Additionally, the diffusion coefficients of both cations and anions were estimated to be approximately 10^−10^ m^2^ s^−1^. This suggested that the characteristic diffusional length scale, 
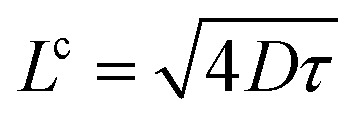
, surpassed considerably the radius of the first solvation shell, *L*^S^. These results strongly indicated a vehicular transportation mode for ion transport within the system.^46^

### Spontaneous formation of monolayer polyelectrolyte-like ion clusters of alkaline earth metal sulfates

**Fig. 3:**
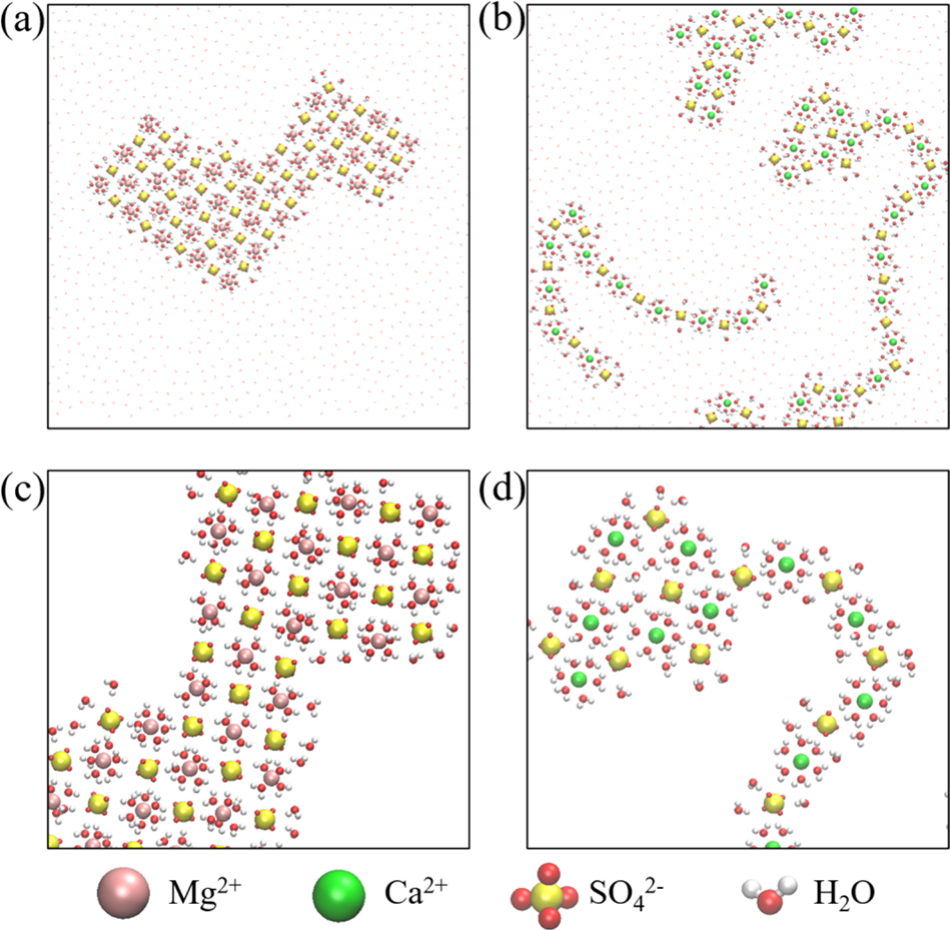
Monolayer polyelectrolytes of alkaline earth metal sulfates in a nanoslit with width *D* = 0.8 nm. Top views of snapshots of (a) a MgSO_4_·6H_2_O/MgSO_4_·4H_2_O hydrated salt and (b) CaSO_4_ polyelectrolyte-like chains. (c and d) The corresponding magnified views of a portion of the systems.

As previously discussed, the self-assembly of monolayer polyelectrolytes with alkali sulfates can be attributed to the delicate interplay between ion dehydration, the Coulomb attractions between monovalent cations and SO_4_^2−^ ions, and the steric constraints within an angstrom-scale slit. To determine whether the SO_4_^2−^ ions can still form monolayer polyelectrolytes when paired with divalent cations, which lead to stronger hydration interactions, we performed additional MD simulations of electrolyte aqueous solutions of MgSO_4_ and CaSO_4_ within the angstrom-scale slit with 0.8 nm width. After equilibration simulations spanning 4–7 μs, we observed the emergence of two types of polyelectrolytes (Fig. 3 and Movies S4, S5†). Notably, the ions remained hydrated by water molecules, indicating that full dehydration did not occur due to the stronger hydration interaction with divalent cations than with monovalent cations.

Fig. 3a illustrates a snapshot of the monolayer hydrated salt of MgSO_4_. In contrast to the monolayer hexagonal honeycomb hydrated salts formed from alkaline-earth chlorides^39^ and the monolayer rhomboidal hydrated salt of 4Na·2SO_4_·6H_2_O, the MgSO_4_ hydrated salt exhibited a square pattern. Each Mg^2+^ ion was hydrated by six (or four) water molecules, and the hydrated Mg^2+^ ions were surrounded by four nearest-neighbour SO_4_^2−^ ions; this structure was hence denoted MgSO_4_·6H_2_O/MgSO_4_·4H_2_O (Fig. 3c). The unit cell configuration differed dramatically from that of monolayer hydrated salts of alkaline-earth chlorides and 4Na·2SO_4_·6H_2_O. The square MgSO_4_ hydrated salt possessed a relatively high packing density due to its small lattice constant of ∼0.47 nm, suggesting the presence of strong Coulomb interactions between the hydrated Mg^2+^ ions and SO_4_^2−^ ions, as in the case of SSIPs. In this context, the square lattice constant was defined as the distance between the nearest neighbour cation–anion within the hydrated salt domains, as indicated by the positions of peaks in the cation–anion RDFs (Fig. S8a†). The coordination number for water molecules around Mg^2+^ ion was smaller than 6 (Fig. S8c†), suggesting the Mg^2+^ ion was surrounded by 6 or 4 water molecules (Fig. 3c).

In stark contrast to the square MgSO_4_ hydrated salt, the Ca^2+^ and SO_4_^2−^ ions spontaneously aggregated into the polyelectrolyte-like chains composed of hydrated ions (Fig. 3b), similar to the Li_2_SO_4_ polyelectrolytes. Notably, square units within the hydrated salt domain were also observed in the electrolyte solution, implying that the formation of hydrated salt domains was energetically favourable. However, Fig. S8b† showed that the square lattice constant of the CaSO_4_ hydrated salt was ∼0.53 nm, slightly larger than that of the MgSO_4_ hydrated salt. And six water molecules surrounded one Ca^2+^ ion (Fig. S8d†). The larger lattice constant resulted in a relatively loose packing density and weaker Coulomb interactions between cations and anions in the CaSO_4_ hydrated salt. Consequently, the square CaSO_4_ hydrated salt was disrupted by water molecules due to thermal fluctuations. In other words, the formation of disordered chain-like polyelectrolytes might be thermodynamically more favourable for CaSO_4_ electrolyte aqueous solutions within the angstrom-scale slit.

### Nonlinear ion conduction under an external electric field

A recent theoretical study showed that the formation of Bjerrum polyelectrolytes under the influence of 2D confinement could induce nonlinear ion transport phenomena, known as the polyelectrolytic Wien (PEW) effect.^31^ And the very recent experimental findings on ion transport in narrow slits,^32^ attributed this nonlinear ion transport phenomenon to broken Bjerrum pairs (Wien effect) or their clustering into conducting arcs (polyelectrolytic Wien effect), under a strong electric field. To determine whether the PEW effect occurs when ions form polyelectrolytes with hydrated ions or anhydrate ionic crystals within an angstrom-scale slit, we performed five additional independent series of NVT MD simulations, each subjected to in-plane (*x*-direction) electric fields, *E*_*x*_, ranging from 0.01 to 1 V nm^−1^. In all the simulations, the initial configurations were chosen as the distinct monolayer polyelectrolytes.

**Fig. 4:**
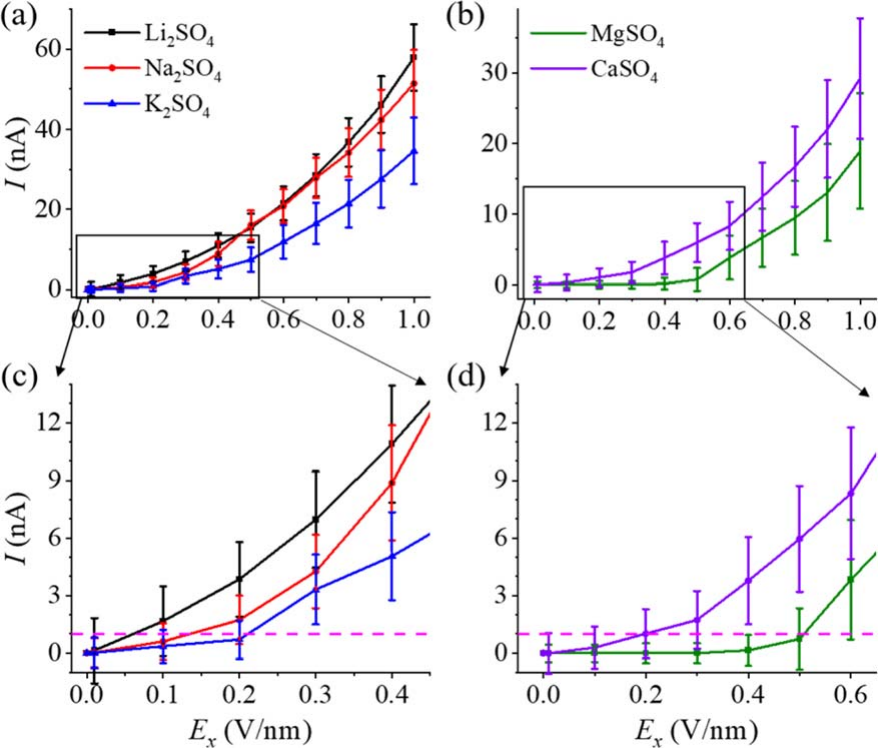
The nonlinear ion conduction of monolayer polyelectrolytes under an external electric field. The *I*–*V* characteristics of aqueous electrolyte solutions in an angstrom-scale slit for (a) alkali sulfates and (b) alkaline-earth metal sulfates. (c and d) Corresponding gaps in the ion conductance of the electric field. The magenta dashed lines in Fig. 4c and d indicate a current of 1.0 nA. The average current was computed using the data from the last 20 ns of each 100 ns simulation under the influence of an electric field.

Fig. 4 displayed the typical current–voltage (*I*–*V*) characteristics of aqueous electrolyte solutions confined within an angstrom-scale slit. Remarkably, the curves for all five polyelectrolytes exhibited a pronounced nonlinear behaviour, regardless of the structure (including chains, hydrated salts, or ionic crystals). At a low electric field, the ion current (*I*) was decreased. Once the electric field exceeded a specific threshold value, the current (*I*) started to increase more rapidly (Fig. 4a and b). Furthermore, the *I*–*V* characteristics of the polyelectrolytes containing divalent ions (Mg^2+^ and Ca^2+^) displayed a more pronounced decrease in the current than polyelectrolytes containing monovalent ions (Li^+^, Na^+^ and K^+^). Additionally, in the Na_2_SO_4_ and K_2_SO_4_ curves, a notable gap in the range of 0.1–0.2 V nm^−1^ was observed, where zero current was detected. However, this gap was absent in the case of the Li_2_SO_4_ system (Fig. 4c). Note that the current values below 1.0 nA were considered zero because the error ranges were larger than 1.0 nA. Similarly, the gap was ∼0.5 V nm^−1^ for the MgSO_4_ system and approximately 0.2 V nm^−1^ for the CaSO_4_ system (Fig. 4d). These results implied that chain-like polyelectrolytes exhibited less current suppression, whereas the hydrated salts and ionic crystals exhibited stronger current suppression, despite having the same valence state of the ions. Note that the observed phenomenon of strong current suppression at low electric fields differs markedly from the phenomenon known as the “PEW effect” reported previously.^31^ In the latter case, the current exhibits a rapid increase at low electric fields (*i.e.*, *I* ∝ *E*^*a*_PEW_(T*)^(*E* → 0), *a*_PEW_(T^*^) = 1 + 1/4T^*^).

**Fig. 5:**
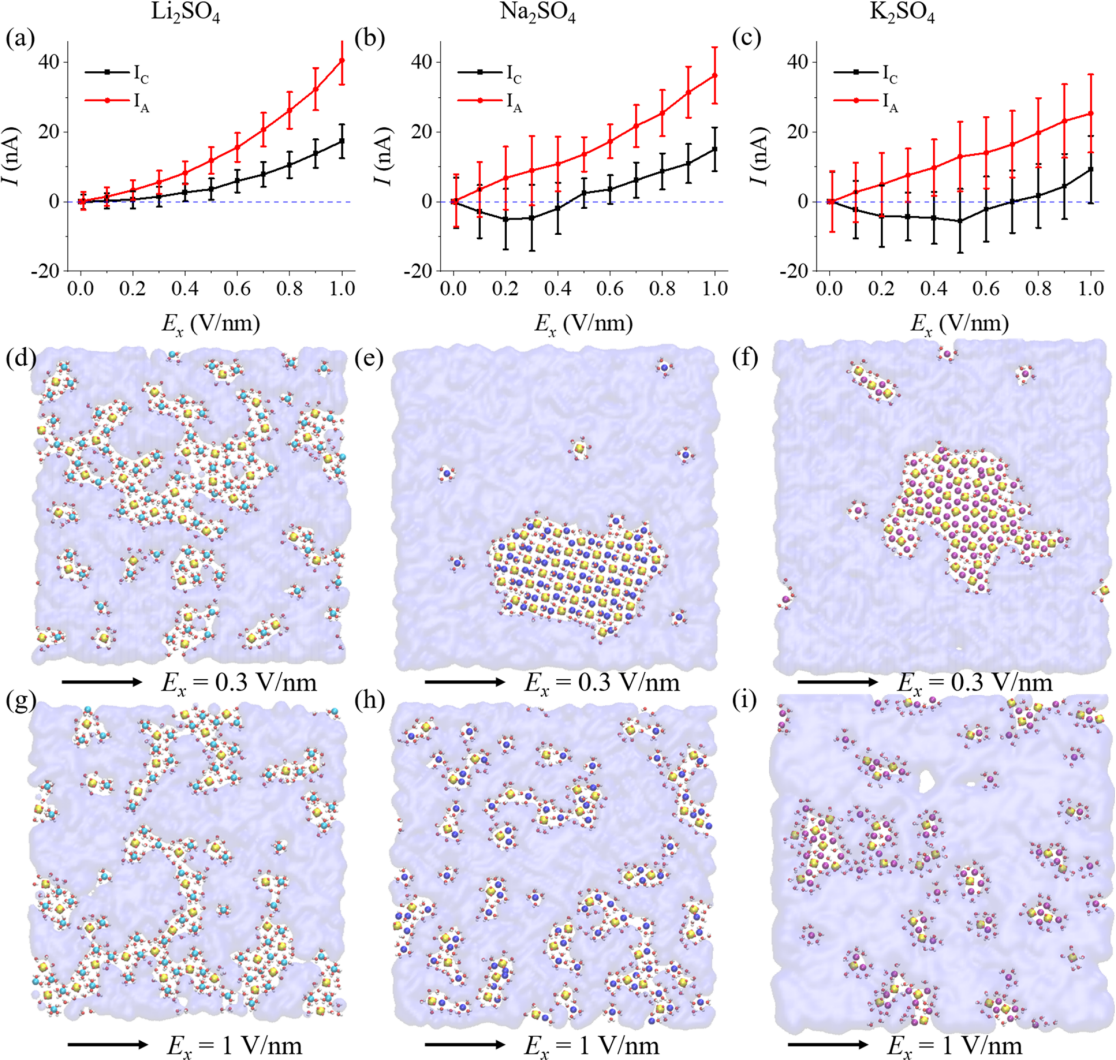
The mechanism of nonlinear ion conductance for monolayer polyelectrolytes of alkali sulfates. Decomposition of the ion current of (a) Li_2_SO_4_, (b) Na_2_SO_4_, and (c) K_2_SO_4_ polyelectrolytes into cationic current (*I*_C_) and anionic current (*I*_A_) components. Corresponding snapshots under *E*_*x*_ = 0.3 V nm^−1^ (d–h) and *E*_*x*_ = 1 V nm^−1^ (g–i), respectively. The blue dashed lines in A–C indicate zero current. The average current was computed using the data from the last 20 ns of each 100 ns simulation under the influence of an electric field.

The nonlinear ion conduction in the angstrom-scale slit deviated from the linear behaviour typically seen in bulk electrolytes. To understand this intriguing nonlinearity, we conducted a detailed investigation into the contributions of cations and anions, breaking down the total current into its cationic and anionic components (Fig. 5 and S9†). Additionally, we explored cluster distributions, effective charges, and the bond density in response to varying electric fields (Fig. S10–S13†), which were commonly studied in bulk electrolytes.^45–48^ Notably, in alkali sulfates (Fig. 5a–c), the anionic current (*I*_A_) consistently exceeded the cationic current (*I*_C_) by a considerable margin.

In the Li_2_SO_4_ system, the polyelectrolyte-like chains exhibited a higher propensity for ion dissolution (see Fig. 5d and Movie S6†). In the absence of an external electric field, both cations and anions aggregated into diverse clusters, akin to polyelectrolyte-like chains, varying in size and charge (Fig. S10a†). As *E*_*x*_ increased, the larger clusters gradually decomposed into free ions and smaller charged clusters (Fig. S10b–d†), consistent with the nonlinear ion conduction behavior (Fig. 4a). Furthermore, Fig. S13a† showed that the cluster bond density of Li_2_SO_4_ chains a gradually decreased with increasing electric field, reflecting dissociation of aggregates into smaller clusters or free ions.

For Na_2_SO_4_ and K_2_SO_4_ polyelectrolytes, a negative cationic current was observed at low electric fields, indicating negative transference number. Upon closer examination of the snapshots (Fig. 5d–f) and dynamic trajectories (Movies S7–S8†), it became evident that at low electric field (*E*_*x*_ ⇐ 0.2 V nm^−1^), the 4Na·2SO_4_·6H_2_O hydrated salt and K_2_SO_4_ ionic crystal remained largely unchanged. Indeed, the distribution of clusters and bond density only showed slight fluctuation (Fig. S11–S13†). Moreover, these extremely large clusters would carry negative charges due to cation dissolution into the aqueous solution (Fig. 5e, f and S11, S12†), facilitating the concerted movement of both cations and anions under the electric field. This led to a positive anionic current and a negative cationic current, resulting in near-zero ion current (*I*) at low electric field, indicative of an ion conductance gating phenomenon. As the external electric field approached 0.3 V nm^−1^, an abrupt change in bond density indicated decomposition of the hydrated salt or ionic crystal into numerous free ions and smaller charged clusters in the solution (Fig. S13b and c†). At *E*_*x*_ = 1.0 V nm^−1^, a significant increase in free ions and smaller charged clusters in solution was observed (Fig. S11d and S12d†), leading to the high ion conduction (Fig. 5g–i and Movies S9–S11†).

The divalent cations, such as Mg^2+^ and Ca^2+^ ions, exhibited stronger Coulomb interactions, resulting in much more stable extremely large clusters. Therefore, much high electric fields were needed to dissociate the polyelectrolytes (Fig. S6†), leading to higher gate fields for the MgSO_4_ and CaSO_4_ polyelectrolytes (Fig. 4) and more pronounced current suppression (Fig. S9†). Hence, the nonlinearity of ion transport largely originated from the formation of polyelectrolyte-like clusters in the absence of an electric field (or at a low electric field) and dissociation under a high electric field.

**Fig. 6:**
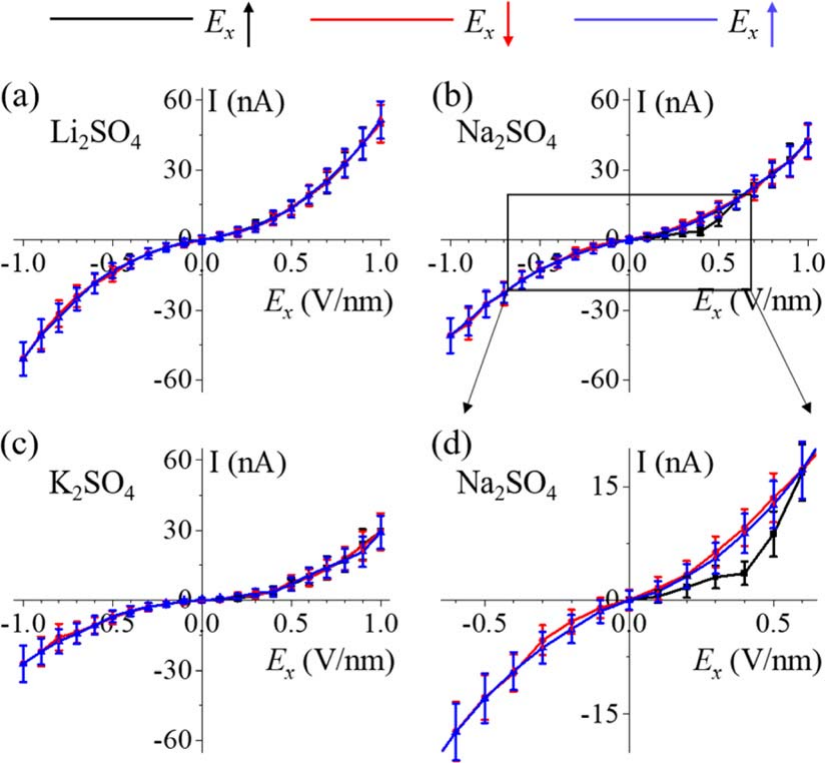
Nonlinear ion conduction at a high frequency. The *I*–*V* characteristics of the aqueous electrolyte solutions in the angstrom-scale slit for (a) Li_2_SO_4_, (b) Na_2_SO_4_, and (c) K_2_SO_4_, respectively. (d) Magnified view of the more pronounced current suppression of the 4Na·2SO_4_·6H_2_O hydrated salt. The average current was computed using the data from the last 5 ns of each 10 ns simulation under the influence of an electric field.

As mentioned above, the nonlinear ion conductance was primarily attributed to the aggregation and dissociation of polyelectrolyte-like ion clusters, which were closely correlated to the molecular timescales. We carried out additional MD simulations to explore how the simulation duration impacted ion transport. In each of the five systems examined, we incrementally varied the electric fields (*E*_*x*_) from 0 to 1 V nm^−1^ and then reversed this process in steps from 1 to −1 V nm^−1^. Finally, we incrementally increased *E*_*x*_ from −1 to 1 V nm^−1^. Each step in these simulations was limited to only 10 ns, effectively resulting in a periodic oscillation of the electric field with a cycle time (T) of 400 ns, equivalent to a high frequency of 15.7 MHz. Remarkably, nonlinearity of the ion conductance was also observed (Fig. 6 and S14†), despite insufficient time for the formation of hydrated salt or anhydrate ionic crystals. This nonlinearity originated from the rapid development of disordered polyelectrolytes (charged clusters) within a few nanoseconds, especially under a low electric field or in the absence of an electric field, which effectively suppressed the ion currents. The results were in agreement with the behaviour of chain-like polyelectrolytes observed for the Li_2_SO_4_ and CaSO_4_ systems (Fig. 4). Specifically, as shown in Fig. 6d, the hysteresis effect highlighted the discrepancy in ion current between the ascending (black line) and descending (red line) phases of the electric field. Notably, for the 4Na·2SO_4_·6H_2_O hydrated salt, the increase in electric field resulted in stronger suppressive effects compared to the case with the decrease in electric field, where disordered polyelectrolytes (charged clusters) exhibited weaker suppressive effects. Accordingly, the hysteresis disappeared as the electric field decreased from 0 to −1 V nm^−1^ and increased from −1 to 1 V nm^−1^, as the hydrated salt was not formed within the relatively short 10 ns simulations.

## Conclusions

In summary, our systematic simulation study demonstrated spontaneous formation of diverse polyelectrolyte morphologies in aqueous solution within angstrom-scale slits. The specific structure of the polyelectrolytes depended on the atomic size and valence state of the ions involved. Notably, we observed that Li_2_SO_4_ and CaSO_4_ assembled spontaneously into polyelectrolyte chains with hydrated ions, while Na_2_SO_4_ and MgSO_4_ formed monolayer hydrated salts, with a rhomboidal pattern for 4Na·2SO_4_·6H_2_O and a square-unit pattern for MgSO_4_·6H_2_O/MgSO_4_·4H_2_O. Both patterns differed from the hexagonal honeycomb pattern observed in monolayer hydrated salts of alkaline-earth chlorides.^39^ Additionally, K_2_SO_4_ adopted a hexagonal honeycomb anhydrate ionic crystal structure. The formation of various polyelectrolytes was attributed to the delicate interplay of enhanced Coulomb interactions, weakened hydration effects and steric constraints within the angstrom-scale slits.

More importantly, we observed nonlinearity of the ion conductance through the angstrom-scale slit under the influence of an electric field. The ion current exhibited a gating behaviour in response to low electric fields for the monolayer hydrated salt and ionic crystal, including 4Na·2SO_4_·6H_2_O, MgSO_4_·6H_2_O/MgSO_4_·4H_2_O and K_2_SO_4_. The mechanism underlying this nonlinearity in ion transport can be explained as follows: The formation of polyelectrolytes facilitated the concerted movement of cations and anions, leading to suppression of the current at low electric field strengths. Once the electric field surpassed a certain threshold value, the current began to increase more sharply because more ions dissolved into the aqueous solution. Overall, our results provide more detailed insights into the nonlinear and voltage-gated ion transport in the angstrom-scale slit and are expected to motivate more research to develop nanofluidic iontronics and ion-based computing.^32,33^

## Methods

### Molecular dynamics simulation

All classical MD simulations were carried out using the GROMACS 2022 package.^49^ The aqueous electrolyte solutions were confined between two parallel hydrophobic smooth walls. The system contained 1222 water molecules, 40 anions (SO_4_^2−^), and 80 monovalent cations or 40 divalent cations. The interactions between the solution and the walls were described by Lennard-Jones (L-J) 10–4 potential functions, which corresponded to the integral of the L-J 12–6 potential characteristic of the graphene walls. The distance between the walls (referred to as the width, *D*) was set at 8 angstroms, allowing the accommodation of monolayer water at ambient pressure. Water was modelled using the TIP4P/2005 model, while ions were represented by the Madrid-2019 model,^50^ which was based on the TIP4P/2005 water model and employed the electronic continuum correction (ECC) method. Notably, the ECC method significantly improved the description of the structural and dynamical properties of electrolyte solutions in comparison to standard approaches.^51–53^ The simulations were performed in the NP_L_T ensemble using periodic boundary conditions in the lateral directions (*x* and *y*). The temperature was maintained at 300 K using a Nosé–Hoover thermostat.^54,55^ The pressure was controlled at 1 bar by means of a Parrinello–Rahman barostat.^56^ A cut-off of 1.0 nm was applied for the Lennard-Jones interactions, and long-range electrostatic interactions were treated with the PME method, with a slab correction to address nonperiodicity in the *z* direction.^57^

In the presence of in-plane (*x*-direction) electric fields *E*_*x*_, the simulations were performed in the NVT ensemble. The electric force acting on a charged atom *i* was determined as *q*_*i*_*E*_*x*_, causing the electromigration of the ions within the confined aqueous solution. The motion of both cations and anions under the applied lateral field (*E*_*x*_) contributed to the generation of an ionic current. The instantaneous ion current, directed along the *x*-axis, was calculated as:1
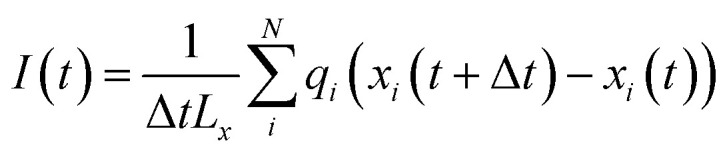
where *x*_*i*_(*t*) represents the instantaneous *x*-coordinate of ion *i*, *L*_*x*_ is the size of the simulation box in the *x*-direction, and Δ*t* denotes the time interval employed for data recording, which was set at Δ*t* = 10 ps.

### Correlation analysis

We computed the transference number utilizing the symmetric mass-transport matrix *L*, based on the theory of transport of correlated systems.^40–44^ The transport coefficient *L*_ab_ was obtained from the slope of the position correlation function (PCF) for the two-dimensional system:2
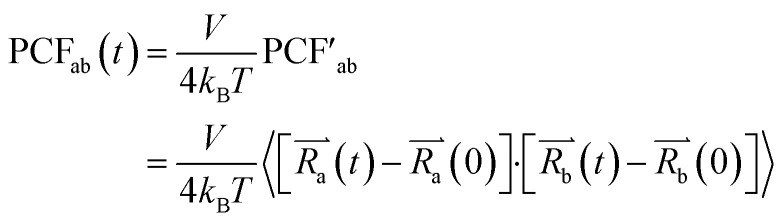
and3
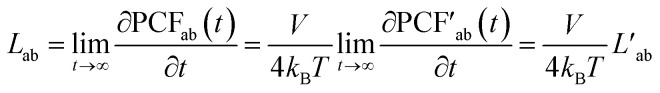
Here, 
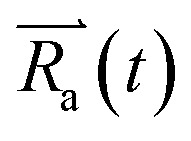
 denoted the vector connecting the center of mass of species “*a*” with that of solvent (*i.e.*, water). Each system comprised three components: cation, anion, and water molecules, resulting in three independent transport coefficients: *L*_CC_, *L*_CA_, and *L*_AA_. The subscript “C” and “A” represented the cation and anion, respectively.

These coefficients can be used to compute ionic conductivity *κ*, and the transference number of *t*_a_:4
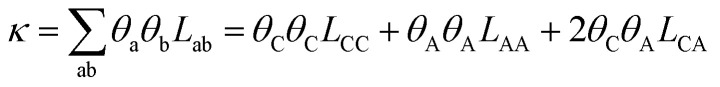
and5
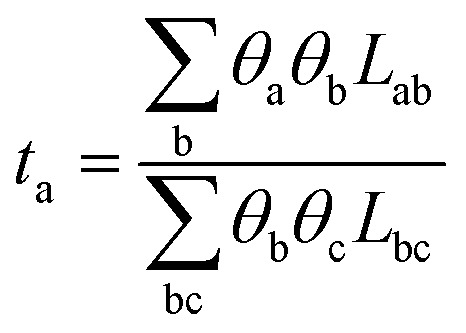
Here *θ*_*i*_ = *Fq*_*i*_*c*_*i*_, where *F* represents Faraday's constant, and *q*_*i*_ and *c*_*i*_ were the charge number and concentration of species “*i*”, respectively. Charge neutrality of the system required *θ*_C_ = −*θ*_A_, thus:6
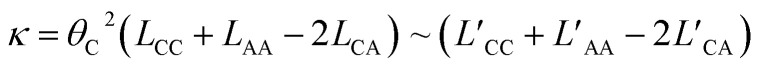
the latter terms are defined as *κ*′;7
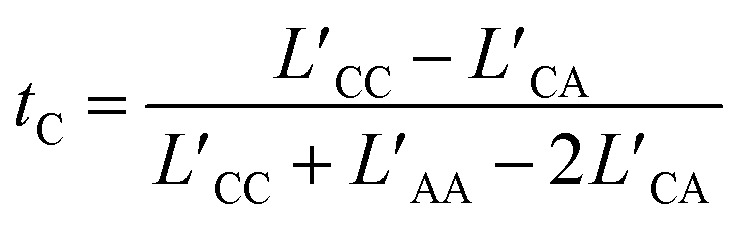
and8
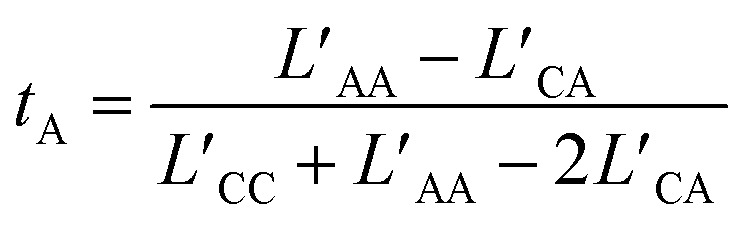


## Data availability

Data are in ESI.†

## Author contributions

X. L. and Y. Z. contributed equally to this work. W. H. Z. and X. C. Z. conceived the project; W. H. Z., X. C. Z. and J. S. F. supervised the MD simulations. X. L., Y. Z. and W. H. Z. performed the MD simulations; W. H. Z., X. L., Y. Z., W. D. Z., W. W. X., X. C. Z. and J. S. F. performed the data analysis; W. H. Z., X. L., Y. Z., X. C. Z., and J. S. F. wrote the manuscript. All authors discussed the results and commented on the manuscript.

## Conflicts of interest

There are no conflicts to declare.

## Supplementary Material

SC-015-D4SC01071J-s001

SC-015-D4SC01071J-s002

SC-015-D4SC01071J-s003

SC-015-D4SC01071J-s004

SC-015-D4SC01071J-s005

SC-015-D4SC01071J-s006

SC-015-D4SC01071J-s007

SC-015-D4SC01071J-s008

SC-015-D4SC01071J-s009

SC-015-D4SC01071J-s010

SC-015-D4SC01071J-s011
