# Supplementary material for: Formation of compounds with diverse polyelectrolyte morphologies and nonlinear ion conductance in a two-dimensional nanofluidic channel
Source: Chem Sci. 2024 May 3;15(21):8170–80. doi: 10.1039/d4sc01071j (PMC11134406; doi:10.1039/d4sc01071j)
Supplement: SC-015-D4SC01071J-s003 [file SC-015-D4SC01071J-s003.pdf]

Electronic Supplementary Material (ESI) for Chemical Science.
This journal is © The Royal Society of Chemistry 2024

Electronic Supplementary Information

Formation of compounds with diverse polyelectrolyte morphologies and nonlinear ion conductance in a two-dimensional nanofluidic channel

Xiaoying Liang,‡[a] Yanan Zhou,‡[b] Weiduo Zhu,[c] Wen Wu Xu,[a] Joseph S. Francisco,*[d] Xiao Cheng Zeng *[e] and Wenhui Zhao*[a]

[a]*Department of Physics, Ningbo University, Ningbo, Zhejiang 315211, China*

[b]*School of Material Science and Chemical Engineering, Institute of Mass Spectrometry, Ningbo University, Ningbo 315211, China*

[c]*Department of Physics, Hefei University of Technology, Hefei, Anhui 230009, China*

[d]*Department of Chemistry, University of Pennsylvania, Philadelphia, Pennsylvania 19104, United States*

[e]*Department of Materials Science & Engineering, City University of Hong Kong, Kowloon 999077 Hong Kong, China*

‡X.L. and Y.Z. contributed equally to this work.

*Authors to whom correspondence should be addressed: zhaowenhui@nbu.edu.cn, xzeng26@cityu.edu.hk, or frjoseph@sas.upenn.edu

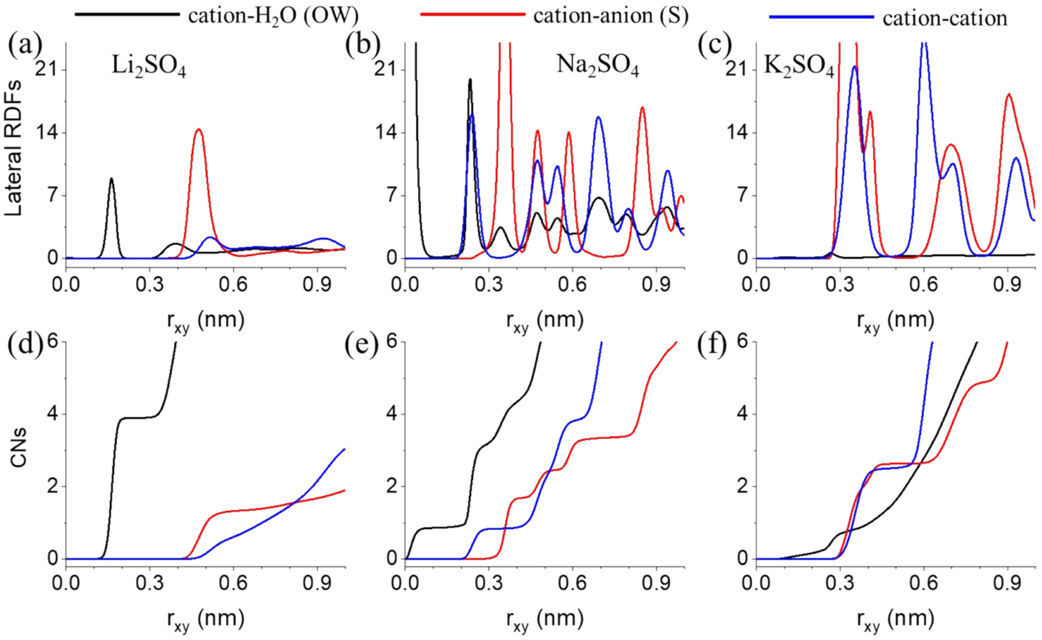

Fig. S1. (a-c) Lateral RDFs of cation-H_2O, cation-cation and cation-anion in the case of (a) Li_2SO_4 polyelectrolyte-like chains, (b) $4Na \cdot 2SO_4 \cdot 6H_2O$ hydrated salt, and (c) K_2SO_4 ionic crystal, respectively. (d-f) Corresponding CNs of the three polyelectrolytes.

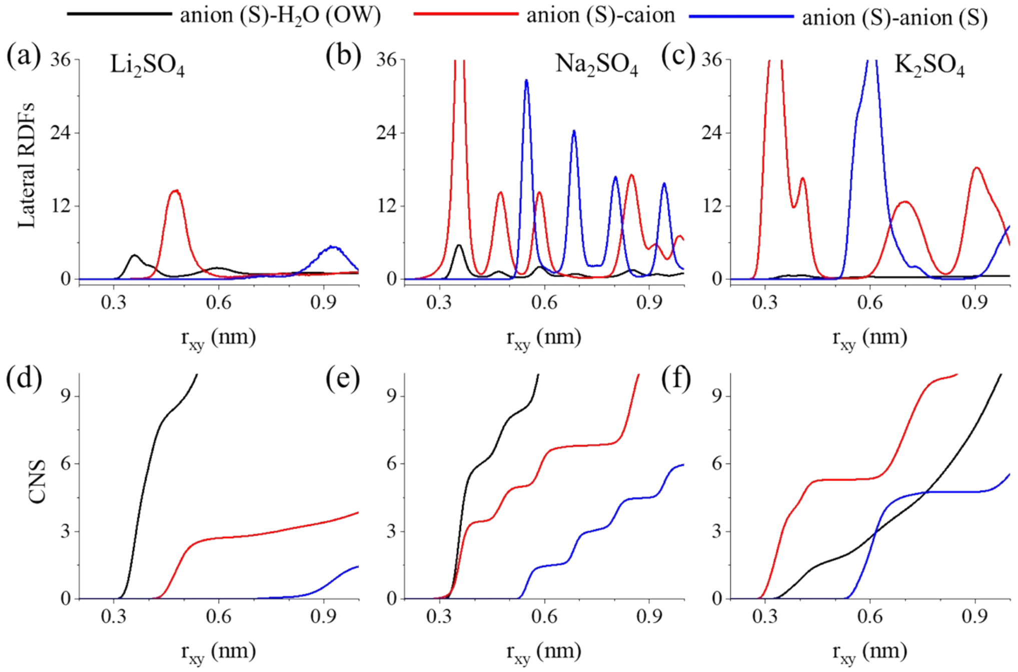

Fig. S2. (a-c) Lateral RDFs of anion-H_2O, anion-cation and anion-anion in the case of (a) Li_2SO_4 polyelectrolyte-like chains, (b) $4Na \cdot 2SO_4 \cdot 6H_2O$ hydrated salt, and (c) K_2SO_4 ionic crystal, respectively. (d-f) Corresponding CNs of the three polyelectrolytes.

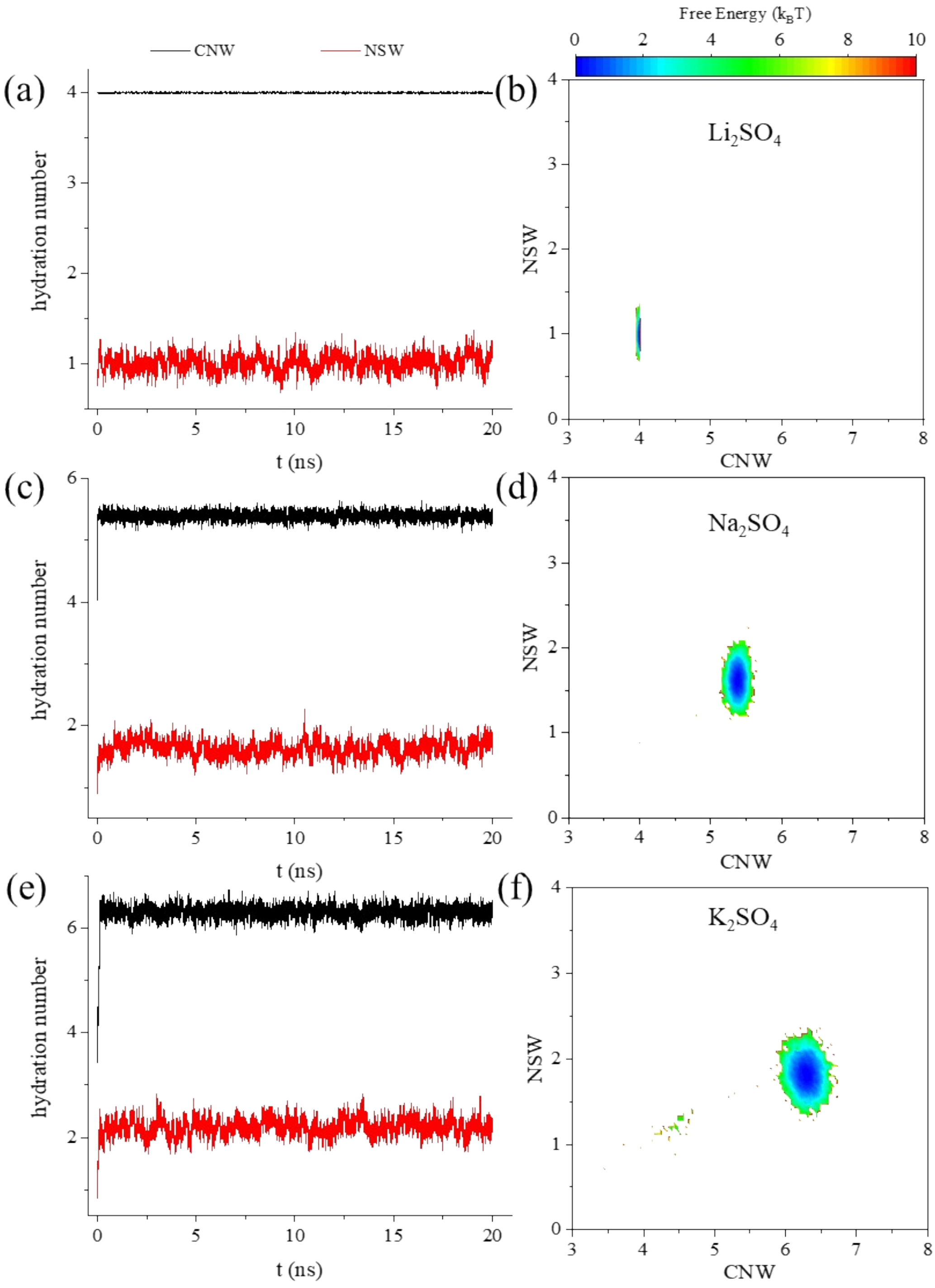

Fig. S3. The hydration number in bulk electrolyte solutions. The time-dependent average coordination number of water molecules (CNW) per cation and the number of shared water molecules (NSW) between cations and anions, computed based on representative snapshots during the 20 ns simulations of (a) Li_2SO4, (c) Na_2SO4 and (e) K_2SO4 aqueous solution in bulk. (b, d, f) The corresponding 2D conformational free energy landscapes.

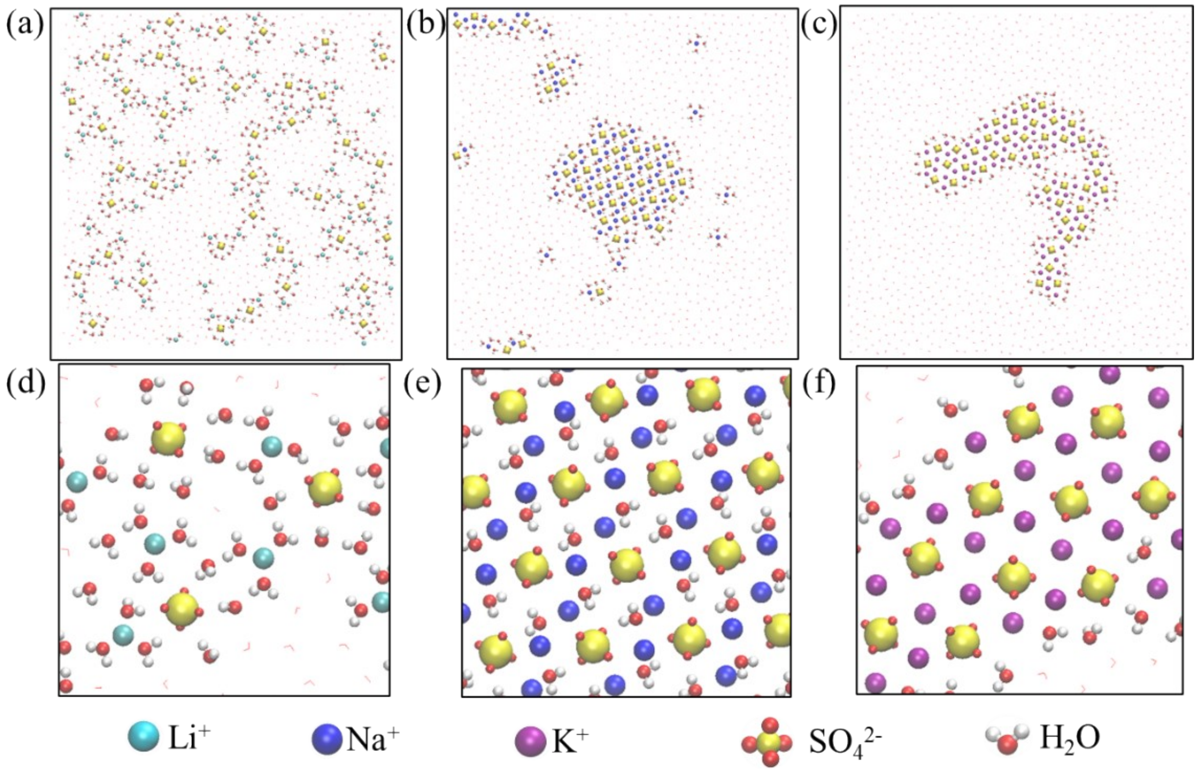

Fig. S4. Monolayer polyelectrolytes of alkali sulfates in the nanoslit with the width *D* = 0.75 nm. Top views of snapshots of (a) Li_2SO_4 polyelectrolyte-like chains, (b) a $4Na{\cdot}2SO_4{\cdot}6H_2O$ hydrated salt, and (c) a K_2SO_4 ionic crystal. (d-f) The corresponding magnified top view of a portion of the snapshots.

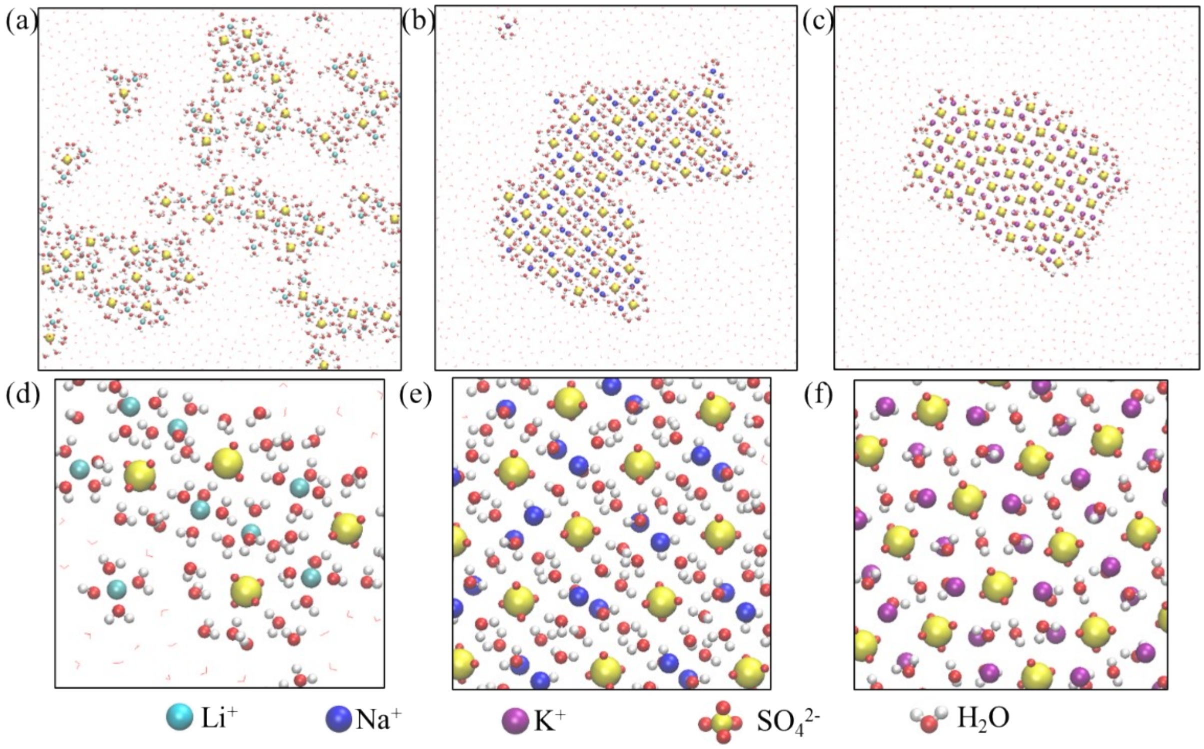

Fig. S5. Monolayer polyelectrolytes of alkali sulfates in the nanoslit with the width D = 0.85 nm. Top views of snapshots of (a) Li_2SO_4 polyelectrolyte-like chains, (b) a Na_2SO_4·hydrated salt, and (c) a $4K{\cdot}2SO_4{\cdot}6H_2O$ hydrated salt. (d-f) The corresponding magnified top view of a portion of the snapshots.

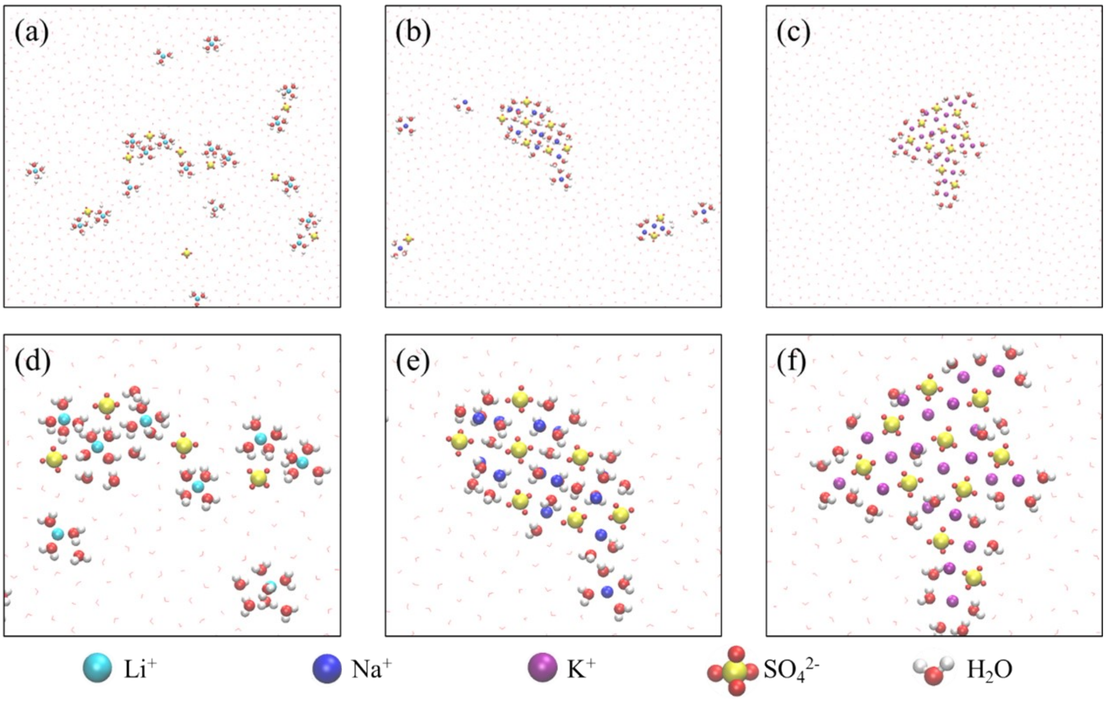

Fig. S6. Monolayer polyelectrolytes of alkali sulfates in the nanoslit with the width $D = 0.8$ nm with only 10 anions and 20 monovalent cations. Top views of snapshots of (a) Li_2SO_4 polyelectrolyte-like chains, (b) a $4Na{\cdot}2SO_4{\cdot}6H_2O$ hydrated salt, and (c) a K_2SO_4 ionic crystal. (d-f) The corresponding magnified top view of a portion of the snapshots.

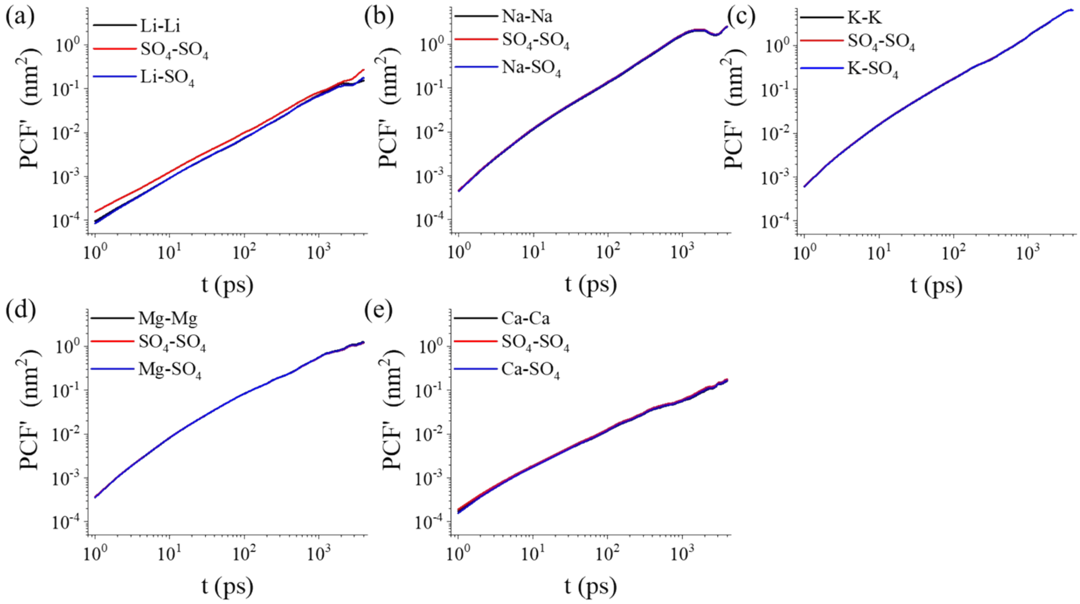

Fig. S7. The PCF' vs time for the (a) Li_2SO_4, (b) Na_2SO_4, (c) K_2SO_4, (d) $MgSO_4$, and (e) $CaSO_4$ aqueous solutions confined in the nanoslit. Here $PCF'_{ab}(t) = \langle [\vec{R}_a(t) - \vec{R}_a(0)] \cdot [\vec{R}_b(t) - \vec{R}_b(0)] \rangle$, and $PCF_{ab}(t) = \frac{V}{4k_B T} PCF'_{ab}(t)$ for the two-dimensional ionic aqueous solution.

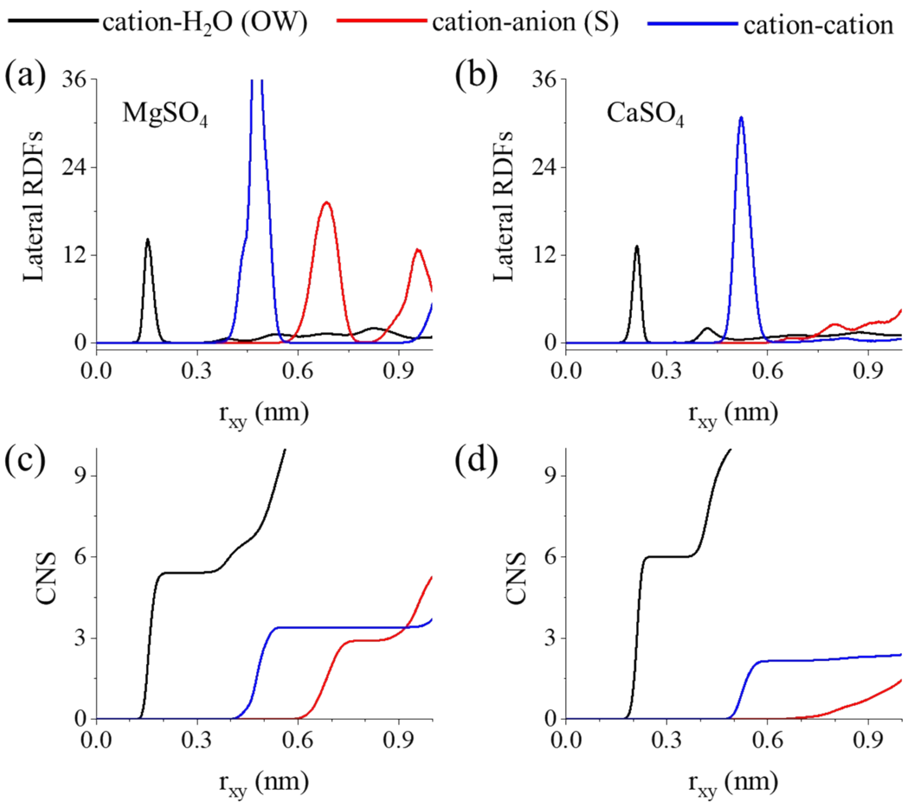

Fig. S8. (a-b) Lateral RDFs of cation-H_2O, cation-anion and cation-cation in the case of (a) $MgSO_4 \cdot 6H_2O/MgSO_4 \cdot 4H_2O$ hydrated salt and (b) $CaSO_4$ polyelectrolyte-like chains, respectively. c-d) Corresponding CNs of the two polyelectrolytes.

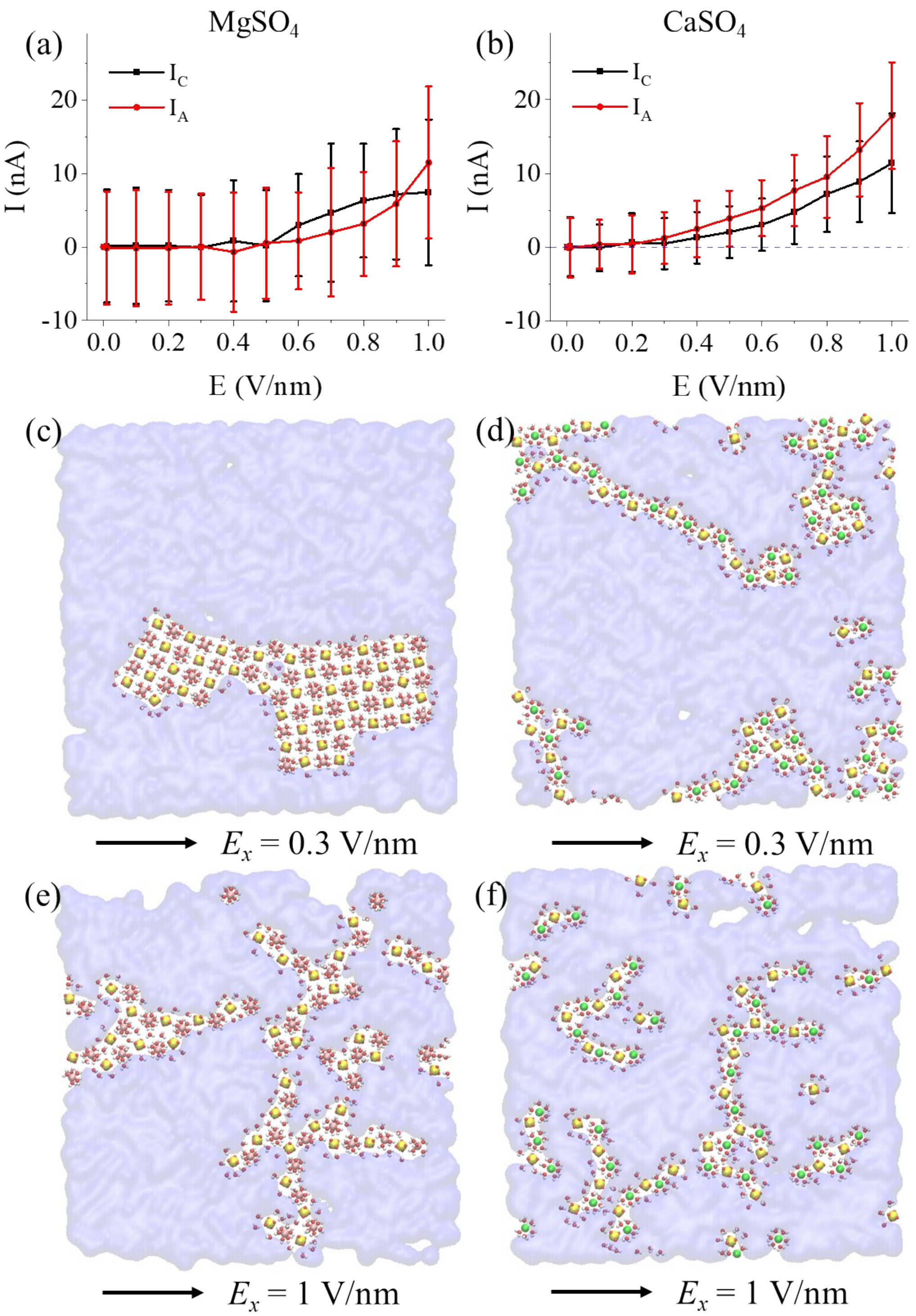

Fig. S9. Decomposition of ion current of (a) $MgSO_4$, and (b) $CaSO_4$ polyelectrolytes to cationic current (I_C) and anionic current (I_A), respectively. Corresponding snapshots under E_x = 0.3 V/nm (c-d) and E_x = 1 V/nm (e-f), respectively.

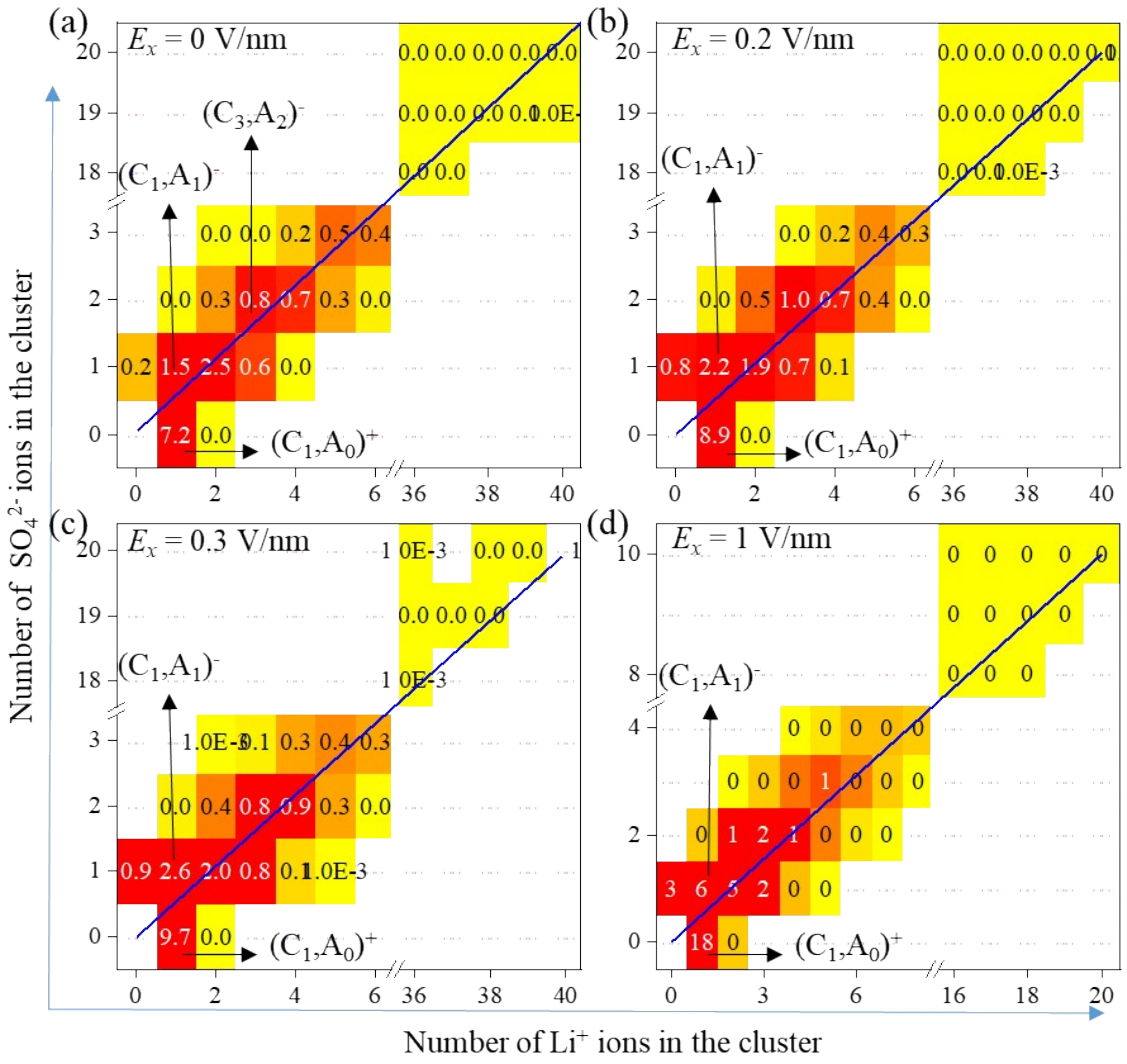

Fig. S10. Cluster distribution of the Li_2SO_4 system as a function of electric field E_x = (a) 0 V/nm, (b) 0.2 V/nm, (c) 0.3 V/nm, and (d) 1.0 V/nm, respectively. The symbol $(C_i, A_j)^{i\text{-}2j}$ represented a cluster comprising *i* Cations and *j* Anions with a charge of *i*-2*j*. The number in the figures indicated the average number of a given cluster per snapshot. The blue oblique line $i = 2j$ suggested the neutral clusters.

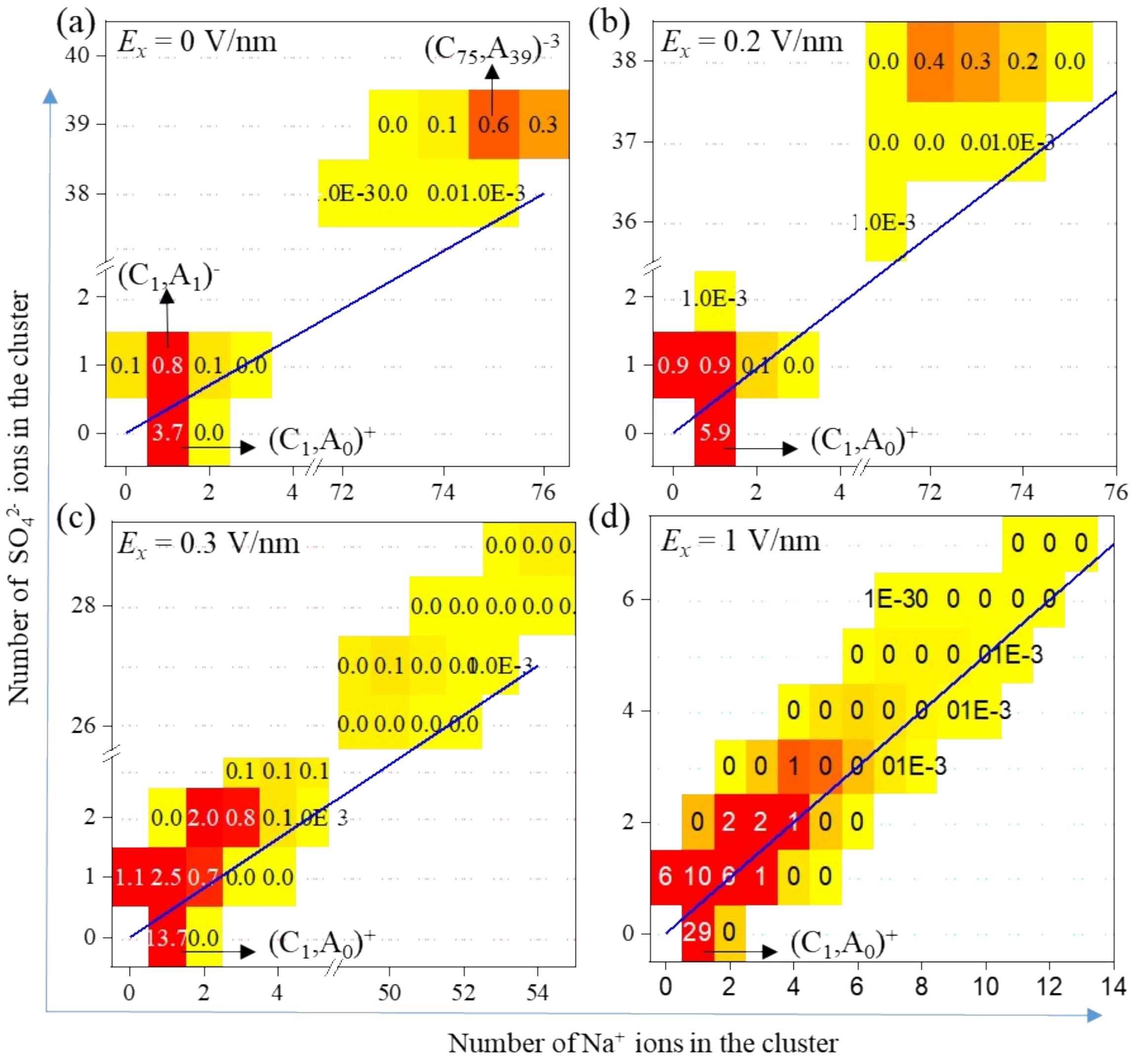

Fig. S11. Cluster distribution of the Na_2SO_4 system as a function of electric field E_x = (a) 0 V/nm, (b) 0.2 V/nm, (c) 0.3 V/nm, and (d) 1.0 V/nm, respectively. The symbol $(C_i, A_j)^{i-2j}$ represented a cluster comprising *i* Cations and *j* Anions with a charge of *i*-2*j*. The number in the figures indicated the average number of a given cluster per snapshot. The blue oblique line *i* = 2*j* suggested the neutral clusters.

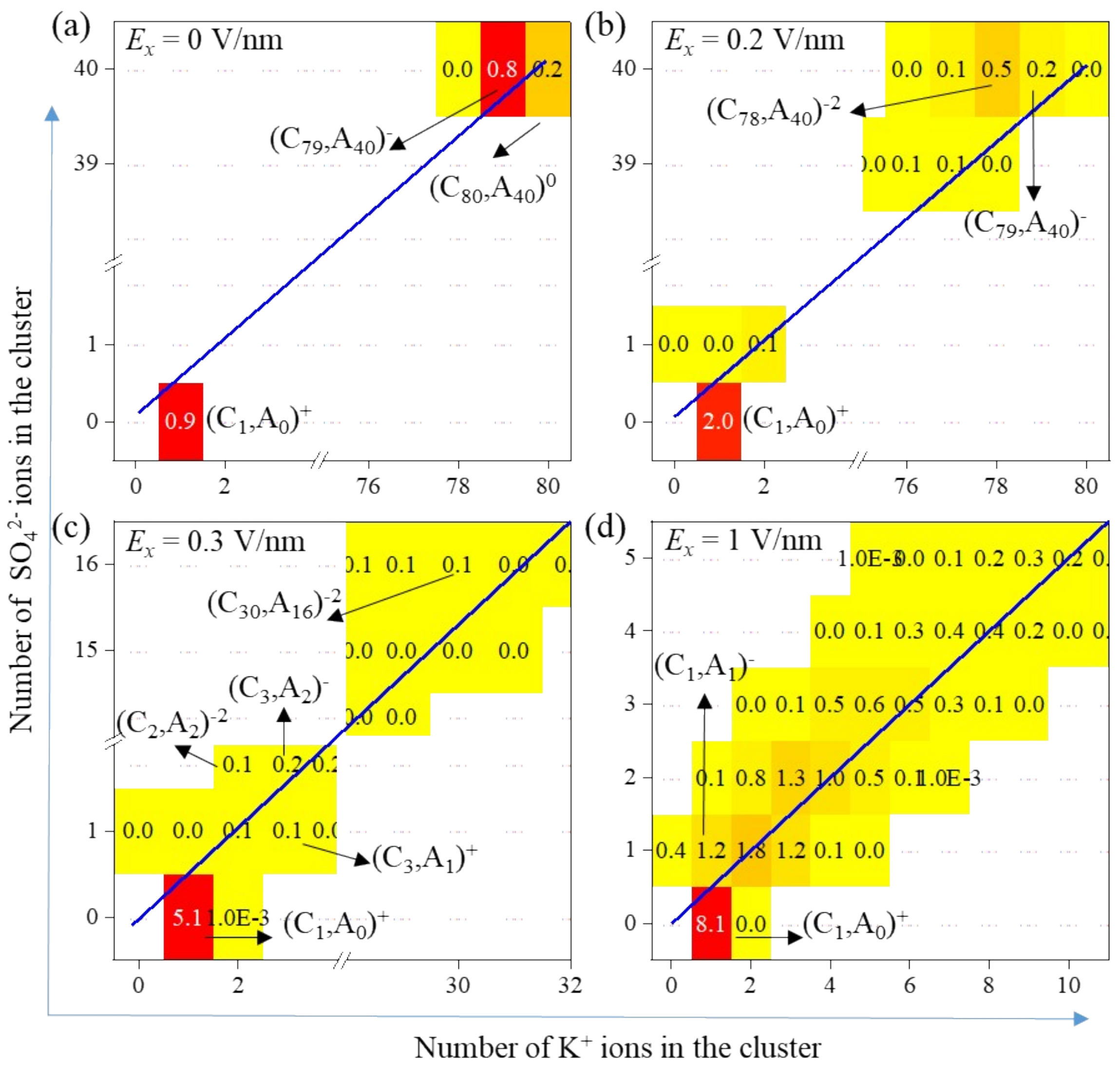

Fig. S12. Cluster distribution of the K_2SO_4 system as a function of electric field E_x = (a) 0 V/nm, (b) 0.2 V/nm, (c) 0.3 V/nm, and (d) 1.0 V/nm, respectively. The symbol $(C_i, A_j)^{i-2j}$ represented a cluster comprising *i* Cations and *j* Anions with a charge of i-2j. The number in the figures indicated the average number of a given cluster per snapshot. The blue oblique line $i = 2j$ suggested the neutral clusters.

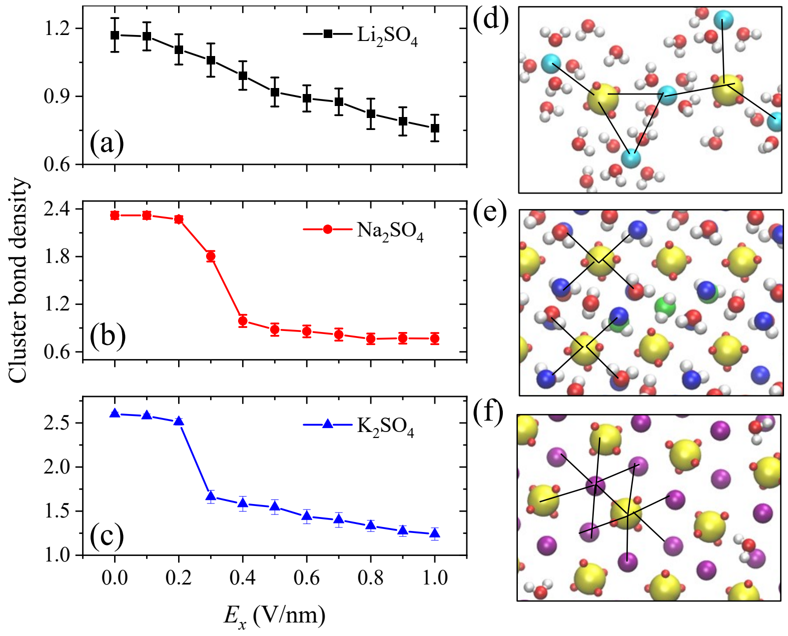

Fig. S13. Cluster bond density in aqueous of (a) Li_2SO_4, (b) Na_2SO_4 and (a) K_2SO_4 as the function of the electric field (E_x). (d-e) The ion associations within the Li_2SO_4 chain, $4Na{\cdot}2SO_4{\cdot}6H_2O$ hydrated salt, and K_2SO_4 ionic crystal in absence of electric field. Here, the associations between ions and water molecules were neglected. The cluster bond density of the Li_2SO_4 chain exceeded 1, primarily due to cation-cation associations.

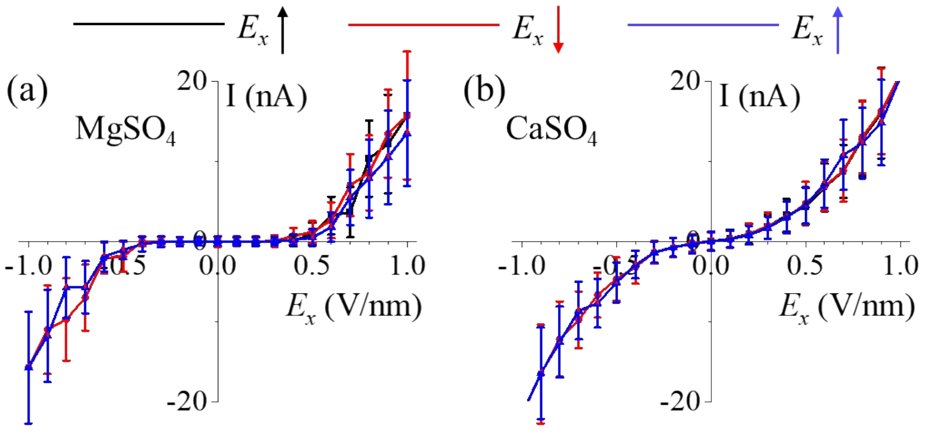

Fig. S14. The *I-V* characteristics of the electrolyte aqueous solutions in the angstrom-scale slit for (a) $MgSO_4$ and (b) $CaSO_4$.
